# Transcriptional profiling of antidepressant ketamine and electroconvulsive therapy treatment

**DOI:** 10.1038/s41398-026-04196-y

**Published:** 2026-08-05

**Authors:** Artemis Zavaliangos-Petropulu, Ginny Ghang, Toni Boltz, Paloma Pfeiffer, Lingyu Zhan, Brandon Taraku, Eliza Congdon, Randall T. Espinoza, Katherine L. Narr, Roel A. Ophoff

**Affiliations:** 1 Ahmanson-Lovelace Brain Mapping Center, Department of Neurology, Geffen School of Medicine at the University of California, Los Angeles, CA USA; 2 Department of Human Genetics, David Geffen School of Medicine, University of California Los Angeles, Los Angeles, CA USA; 3 Jane and Terry Semel Institute for Neuroscience and Human Behavior, Department of Psychiatry and Biobehavioral Sciences, Geffen School of Medicine at the University of California, Los Angeles, CA USA

**Keywords:** Depression, Pharmacogenomics

## Abstract

**Background:**

Treatment-resistant depression (TRD) affects 30–50% of patients with major depressive disorder (MDD). Electroconvulsive therapy (ECT) and sub-anesthetic ketamine treatment can relieve TRD, yet their antidepressant mechanisms remain unclear. We hypothesized that peripheral blood gene expression might offer a non-invasive proxy of potential treatment-response biomarkers.

**Methods:**

We conducted a transcriptome analysis on peripheral blood samples from individuals with TRD undergoing ECT (N = 37) or serial ketamine infusions (N = 60), and non-depressed controls (N = 35). Samples were collected at baseline and at multiple follow-up time points. Differential gene expression (DGE) at the single gene and network level identified transcriptional changes and co-regulated gene modules associated with diagnosis, treatment, and response/remission status using Weighted Gene Co-Expression Network Analysis (WGCNA), correcting for multiple comparisons.

**Results:**

Longitudinal transcriptional changes were not detected for either treatment for individual genes or networks (FDR corrected or |logFC| >0.05). When comparing remitters and non-remitters at baseline in the ketamine group, we observed evidence of enrichment for immune-related functions overall with one gene significantly differentially expressed (i.e., IGKV1-9) (p = 2.5E-05, logFC = −0.51). In the ECT sample, when considering gene networks, we observed significant interaction effects between time and diagnosis. At least six co-regulated gene modules yielded significant differences at baseline between ECT patients and controls.

**Conclusion:**

Despite the robust clinical improvements associated with ECT and ketamine, peripheral blood RNA-seq revealed only limited longitudinal gene expression changes. However, pre-treatment differences in gene expression profiles suggest some potential predictive value. Larger samples may be warranted to detect possible peripheral molecular signatures of rapid-acting antidepressant response.

## Introduction

Major depressive disorder (MDD) is a debilitating psychiatric disorder, affecting over 250 million people worldwide [1]. Despite advancements in pharmacological and psychotherapeutic interventions, a significant proportion of patients—between 30–50%—do not benefit from conventional treatments, a condition known as treatment-resistant depression (TRD) [2–4]. TRD poses considerable challenges [5], including increased risks of chronic illness [6], disability [7], and suicidal behavior [8], necessitating alternative therapeutic strategies.

Electroconvulsive therapy (ECT) [9–11] and ketamine [12, 13] are both rapid-acting alternative treatment options for patients with TRD. ECT, which induces a brief seizure through electrical stimulation of the brain, has long been considered amongst the most effective interventions for severe depression [14]. Ketamine, traditionally used as an anesthetic, has since been recognized for its rapid and pronounced antidepressant effects when administered in subanesthetic doses [15]. Despite progress [16, 17], the molecular mechanisms underlying ECT and ketamine’s antidepressant effects remain largely unknown.

Preclinical brain-derived gene expression studies have provided important insights into the molecular mechanisms potentially underlying the antidepressant effects of ECT [18–20] and ketamine [21–23]. Unfortunately, experiments requiring access to brain tissue in living humans are not feasible. However, although the brain and peripheral blood exhibit distinct transcriptional profiles [24], prior data shows a meaningful overlap in the expression of genes implicated in depression, suggesting that more accessible sampling of peripheral blood may also reveal CNS mechanisms [25–27]. For instance, findings suggest that 35–80% of known transcripts are detectable in both brain and blood tissue samples [25], supporting the utility of using peripheral blood as a proxy for some aspects of brain gene expression. Several depression-related genes and pathways, particularly those involved in inflammation and immune regulation, are similarly dysregulated in brain tissue and peripheral blood [28–31]. This overlap provides further rationale for using peripheral blood to investigate gene expression changes that, while potentially distinct in the brain, may still reflect biomarkers and treatment response mechanisms in psychiatric disorders.

Few prior studies have reported changes in peripheral blood gene expression with antidepressant treatment, primarily focusing on selective serotonin reuptake inhibitors (SSRIs) [32–36]. However, these studies show limited overlap in identified genes and have yielded inconsistent findings across cohorts, restricting their generalizability. For example, a large study by Nøhr et al. [37], which included 281 patients in a placebo-controlled trial of vortioxetine (n = 97 placebo), found no significant longitudinal changes in gene expression after 8 weeks of treatment. While some baseline gene expression markers were modestly correlated with treatment response, overall effects were small. The authors interpreted these results to imply that antidepressant response may exert subtle effects across many genes, such that even relatively large sample sizes may be underpowered to detect these distributed signals. However, SSRIs require several weeks to exert clinical effects, during which time numerous external and internal factors may contribute transcriptional noise.

Since ECT and ketamine both exhibit rapid and robust behavioral effects, these treatment modalities present a valuable opportunity to evaluate peripheral blood gene expression molecular signatures linked with outcomes and brain-derived phenotypes. Preclinical studies suggest that ketamine’s rapid effects are mediated through N-methyl-D-aspartate (NMDA) receptor antagonism, leading to enhanced glutamatergic transmission, increased brain-derived neurotrophic factor (BDNF) release, and downstream activation of pathways supporting synaptogenesis and dendritic remodeling [38]. ECT, while mechanistically distinct, is also associated with widespread neuroplastic changes, including synaptic remodeling and altered connectivity across mood-relevant brain networks [39]. Both interventions have additionally been linked to modulation of inflammatory and immune signaling pathways [40–43], raising the possibility that peripheral blood may capture aspects of their molecular effects. However, the extent to which these central mechanisms are reflected in peripheral blood remains unclear, as few human studies have directly examined transcriptomic changes following rapid-acting antidepressant treatment [44–46]. Israel-Elgali et al. [45] reported significant ECT-related changes specifically in peripheral blood mononuclear cells (PBMCs) in a sample of 17 participants undergoing ECT. In contrast, Ryan et al. [44] found no significant longitudinal changes in peripheral blood mRNA following ECT in 88 participants, although they did observe pre-treatment differences between patients and controls. Similarly, Cathomas et al. [46] identified primarily cross-sectional differences in gene expression, both between patients with TRD (N = 26) and unaffected controls (N = 21), and between ketamine responders and non-responders.

Here, we performed a whole-blood transcriptome analysis (using RNA sequencing) of patients with TRD undergoing either naturalistic, open-label ECT or ketamine treatment. The overall goal was to identify differential gene expression (DGE) genes and molecular pathways associated with rapidly acting antidepressant response for either treatment modality. We hypothesized that given the robust and rapid clinical effects of ECT and ketamine, peripheral blood would show measurable transcriptional changes associated with treatment response, potentially revealing blood-based biomarkers of antidepressant efficacy. By exploring the transcriptional landscape in peripheral blood, we expected to identify gene changes that would elucidate the mechanisms of rapid therapeutic response and identify blood-based biomarkers that could potentially inform treatment outcomes, thereby contributing to the ongoing efforts to personalize interventions for depression.

## Methods

Data for this study were collected under a NIMH-supported Connectomes Related to Human Disease (CRHD) U01 grant (MH110008, Clinical Trial: NCT02165449) [47]. The primary aim of the project was to identify connectome-based imaging correlates and predictors of treatment response to three rapid-acting antidepressant interventions: ECT, serial ketamine, and total sleep deprivation. Peripheral blood for RNA-sequencing was collected as a secondary objective, using two PaxGene RNA tubes per blood draw at set time points. Tubes were incubated at room temperature for 2 h, then frozen and transported to the UCLA Pathology Research Portal for long-term storage, pending batch extraction and quality control by the UCLA Neuroscience Genomics Core.

### Participants

Patients receiving ketamine and healthy control participants were recruited from the Los Angeles area through advertisements or clinician referral, or clinicaltrials.gov (NCT02165449) for ketamine specifically. ECT participants were recruited from individuals already scheduled to receive ECT as part of routine care at the Resnick Neuropsychiatric Hospital at UCLA. All patient and control participants received diagnostic evaluation using the Structured Clinical Interview for DSM-5. Inclusion criteria for all subjects recruited were age between 20–64 years, able to read and write English and provide informed consent. Exclusion criteria for all participants were dementia, schizophrenia spectrum disorders, or a psychotic disorder due to a general medical condition, neurological disorder, or other serious medical condition, current or history of substance abuse (within the last three months) and pregnancy, each confirmed by blood and urine laboratory tests. TRD patients receiving Ketamine or ECT were recruited independently (not randomized), though their inclusion/exclusion criteria were similar as described below. Table 1 provides demographic and clinical information for each sample at baseline.

**Table 1:** Demographics for the ketamine and ECT samples and healthy controls (HC), with each treatment group compared with HCs separately.

	TRD	HC	P-value
*Ketamine*			
N	60	35	-
Age	39.5 ± 11.2	33.5 ± 12	0.019
Sex (Percent female)	46.7%	54.3%	0.6
Baseline HDRS	18.8 ± 4.75	-	-
Duration of Illness (years)	3.9 ± 5.9	-	-
*ECT*			
N	37	35	-
Age	41.6 ± 16.0	33.5 ± 12	0.018
Sex (Percent female)	67.6%	54.3%	0.36
Baseline HDRS	21.3 ± 6.7	-	-
Duration of Illness (years)	2.9 ± 6.9	-	-

Additional information about race, education, concurrent medications, and other psychiatric comorbidities can be found in Supplemental Tables 2–3.

#### Ketamine participants

Participants receiving ketamine met DSM-5 criteria for a major depressive episode (MDE) (unipolar or bipolar depression with/without psychotic features) and TRD, defined as not having achieved adequate response following ≥2 antidepressant trials of sufficient dosage for > 4–6 weeks each and having experienced continuous depression for >6 months. Inclusion required moderate to severe baseline depressive symptoms (17-Hamilton Depression Rating Scale (HDRS) score of 17 or higher), and stable use of antidepressants or mood stabilizers for >6 weeks before enrollment and throughout the course of the study. Additional exclusion criteria included history of seizures or withdrawal-related convulsions, prior neuromodulation treatment or ketamine within the last six months, past psychotic reactions to medications and any other contraindications to ketamine. Patients were also required to taper from any use of benzodiazepines (as judged by psychiatrist) >24 h prior to treatment sessions.

#### ECT participants

Eligibility for ECT itself was independently verified based on clinical indications according to the APA ECT Task Force Report guidelines. ECT treatment protocols were not manipulated and was provided as clinically indicated. Inclusion and exclusion criteria were otherwise the same as for the ketamine group. However, ECT patients discontinued medications, including antidepressants and anticonvulsants, for at least 48 to 72 h prior to initiation of ECT and study enrollment.

#### Controls

Unaffected controls did not receive any treatment. A subset of control subjects had data collected at a second time point within a similar time frame as the post-fourth infusion ketamine time point (2 weeks). Additional exclusion criteria for controls were any current or past psychiatric disorders or use of any antidepressants or mood stabilizers within the past 6 months. All participants gave written informed consent in accordance with procedures approved by the UCLA Institutional Review Board (IRB).

### Study design

#### Ketamine treatment

**Fig. 1:**
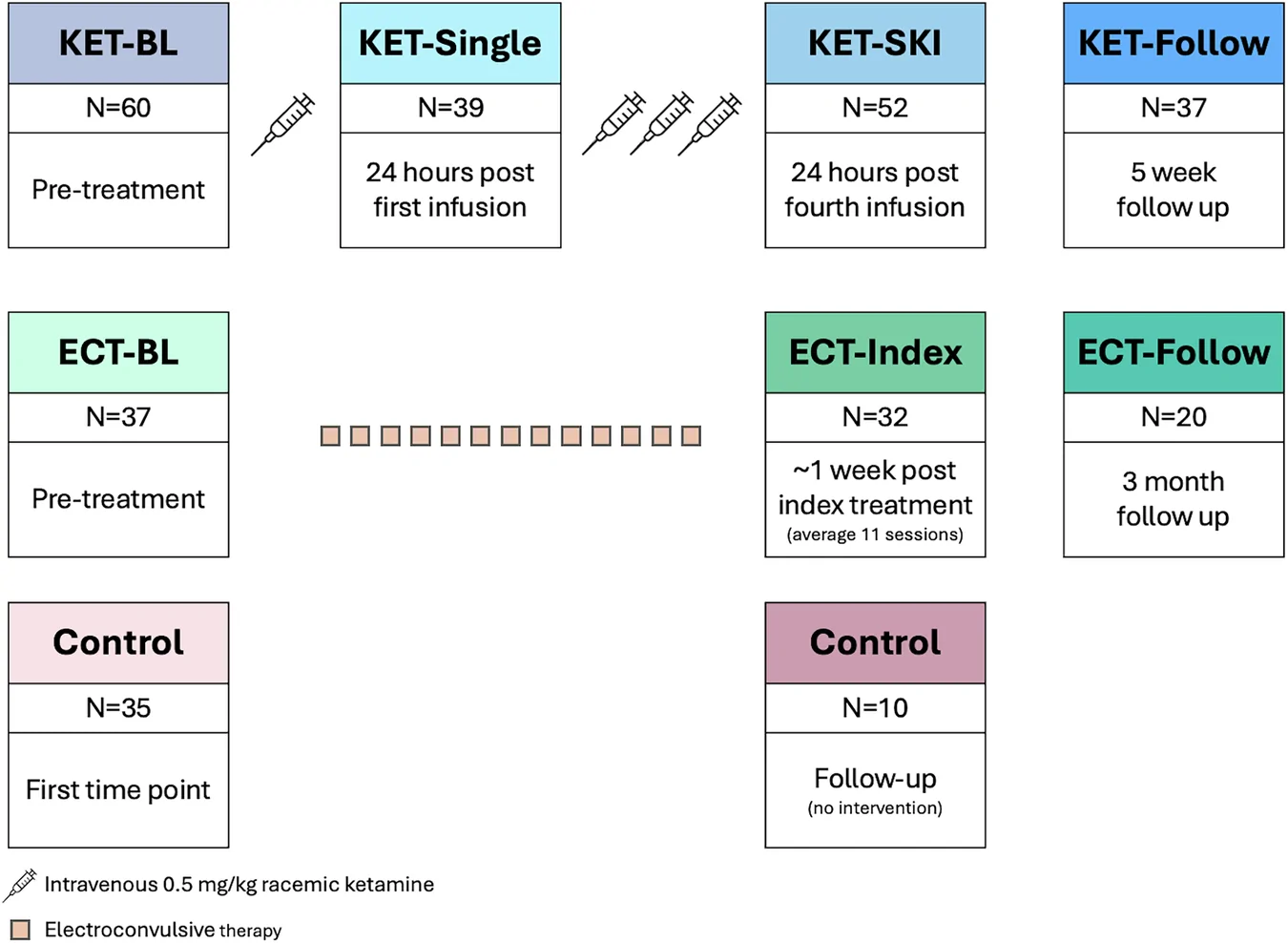
Study design for ketamine (KET) and ECT blood data collection, including sample sizes and description of time points. Demographics for full sample can be found in Table 1, and different sample sizes across time points can be found in Supplemental Table 1.

Using an open-label design, patients received an intravenous infusion of racemic ketamine (0.5 mg/kg) administered over 40 min, four times over two weeks. Blood was collected at baseline, 24 h following the first infusion, 24 h following the fourth infusion, and at a 5-week follow-up (Fig. 1). All controls had blood collected at baseline and a subset of controls had blood collected again two weeks later.

#### ECT treatment

Using a naturalistic design, eligible patients received a clinically prescribed course of ECT (5000Q MECTA Corp., Tualatin, Oregon), administered three times per week. Anesthesia and muscle relaxation were achieved using methohexital (1 mg/kg) and succinylcholine (1 mg/kg). ECT followed the seizure threshold (ST) titration method. Right-unilateral (RUL) ECT was delivered at five times the ST with an ultrabrief pulse width (0.3 ms), while bilateral ECT was given at 1.5 times ST with a brief pulse width (0.5 ms). All patients initially received RUL ECT, but ~30% transitioned to bilateral lead placement based on clinical judgement of response rates. On average, patients underwent 10.9±3.9 ECT sessions, with an average ST of 29.57±25.94 mC and an average seizure duration of 67.8±19.5 s across all ECT treatments. Patients were evaluated at three time points: within 24 h before the first ECT session (baseline), within one week of completing the ECT index series occurring ~3–4 weeks after treatment initiation, and three months after ECT treatment ended or symptom relapse, whichever occurred first (Fig. 1).

### RNA-sequencing

RNA sequencing was performed by the UCLA Neuroscience Genomics Core using an Illumina sequencer, with library preparation conducted using the TruSeq + RiboZero Gold protocol. RNA-seq data quality was visually assessed using FastQC(32), which provided comprehensive quality control metrics for each sample, including per-base sequence quality, GC content, and adapter contamination. Samples failing initial quality checks were excluded from the analysis. Kallisto [48] was used to pseudoalign reads to the GRCh38 gencode transcriptome and quantify estimates of transcript expression. Samples with RNA Integrity Number (RIN) < 6 were excluded. Genomic data for this study can be found in the Gene Expression Omnibus database repository.

### Data analysis

Kallisto outputs were read into R version 4.3.0 gcc 10.2.0 and analyzed as transcripts per million (TPM). We filtered out non–protein-coding genes and genes <10 reads per million mapped in at least two libraries, resulting in a different number of genes analyzed for each dataset due to varying sample sizes (Table 2). TPM values were (log2 + 1)-transformed and analyzed using *limma-voom* [49] to perform differential gene expression (DGE) analysis, which tests whether individual genes show statistically significant differences in expression levels between groups or across time points. *DuplicateCorrelation* was used to adjust for within-subject variability when performing longitudinal analyses. The analysis yielded similar results when re-run using estimated counts (via *tximport*) and DESeq2 [50], indicating that the limited number of differentially expressed genes is not driven by TPM-based normalization. R-package *WGCNA* [51] was used to perform Weighted Gene Co-Expression Network Analysis (WGCNA), a dimensionality reduction approach that groups genes into modules based on shared expression patterns. This method allows genes to be studied as networks rather than in isolation, reflecting the fact that biological processes are typically driven by coordinated activity across sets of genes. A minimum module size of 30 was used for conducting WGCNA. Multidimensional scaling (MDS) plots, mean-variance j-plots, and volcano plots can be found in Supplemental Material. Code used for data analysis available upon reasonable request.

**Table 2:** Summary of DGE findings.

	Genes analyzed after filtering	FDR(0.05) corrected	|LogFC| >0.5
*KETAMINE*			
Baseline-Single	7331	0	0
Baseline-Serial	7465	0	0
Baseline-Follow up	7337	0	0
Time*Remission	7465	0	1 UP, 2 DOWN
Time*Diagnosis	7499	0	1 UP, 2 DOWN
Baseline Remitters vs Non-Remitters	6812	1 UP, 4 DOWN	1 UP, 4 DOWN
Baseline TRD vs HC	7670	28 UP, 36 DOWN	4 UP, 3 DOWN
*ECT*			
Baseline-Index	8300	1 UP 1 DOWN	3 UP, 1 DOWN
Baseline-Follow up	7811	0	0.
Time*Responder	8300	0	0
Time*Diagnosis	8314	0	1 UP, 1 DOWN
Baseline Responders vs Non-Responders	7954	0	4 UP, 5 DOWN
Baseline TRD vs HC	8007	6 UP, 12 DOWN	1 UP, 6 DOWN

The number of genes analyzed and their significance thresholds differ for pairwise comparisons between baseline-single infusion, baseline-serial infusion, and remitters vs non-remitters at baseline.

#### Differential Gene Expression (DGE)

##### Ketamine DGE

We performed pairwise comparisons across time points in patients receiving ketamine treatment, adjusting for age, sex, RIN and the first 10 principal components (PC). Pairwise comparisons included (1) baseline to post-first (single) infusion, (2) baseline to post-fourth infusion and (3) baseline to follow-up. To evaluate effects of remission status, remitters were classified as patients achieving a post-fourth infusion HDRS score of ≤7. A mixed-effect model tested for time*remission interaction by comparing baseline to post-fourth infusion. We similarly tested for a time*diagnosis interaction including controls with longitudinal data. We also performed a DGE analysis comparing baseline gene expression in remitters compared to non-remitters, and patients compared to controls. All analyses were corrected for multiple comparisons using False Discovery Rate (FDR(0.05)) [52]. Functional classification of findings was determined using Gene Ontology for results with log fold change (logFC) ≥ 0.5 or ≤–0.5 (corresponding to a ≥ 1.4-fold change) [53], serving as an effect size filter to exclude very small shifts and focus on changes more likely to be biologically meaningful.

##### ECT DGE

Similar to the ketamine analysis, we performed pairwise comparisons across time points in patients receiving ECT, adjusting for age, sex, RIN and the first 10 PCs. ECT pairwise comparisons included 1) baseline to post-index and 2) baseline to follow-up. Clinical response was defined as a ≥ 30% reduction in HDRS scores post-index, following the widely used criterion for indicating clinically meaningful response [54]. Time*remission and time*diagnosis models included baseline and post-index time points.

#### Network analysis

WGCNA modules were generated using all data points with RIN ≥ 6 from the controls, ketamine, and ECT samples. Eigengenes for each module were calculated for individual samples. In the ketamine sample, limma was used to test for associations between eigengenes and time, time*remission interaction, time*diagnosis, and remission status and diagnosis at baseline. Likewise, in the ECT sample, limma was used to test for associations between eigengenes and time, time*response, and time*diagnosis interactions, and response status and diagnosis at baseline.

## Results

Sixty-five participants were enrolled for ketamine infusions, but after filtering for RIN, N = 60 were analyzed. By the fourth infusion, 48% of participants receiving ketamine achieved remission (HDRS ≤ 7). Forty-one participants were enrolled for ECT, but after filtering, N = 37 were analyzed. Only 25% of patients achieved remission following ECT, thus, to allow balanced statistical comparisons, we focused on categorizing groups based on extent of treatment response as typically performed in ECT studies. Post-Index ECT, 49% of participants were classified as responders (≥30% decrease in HDRS from baseline to post-index).

### DGE analysis

#### Ketamine DGE analysis

**Fig. 2:**
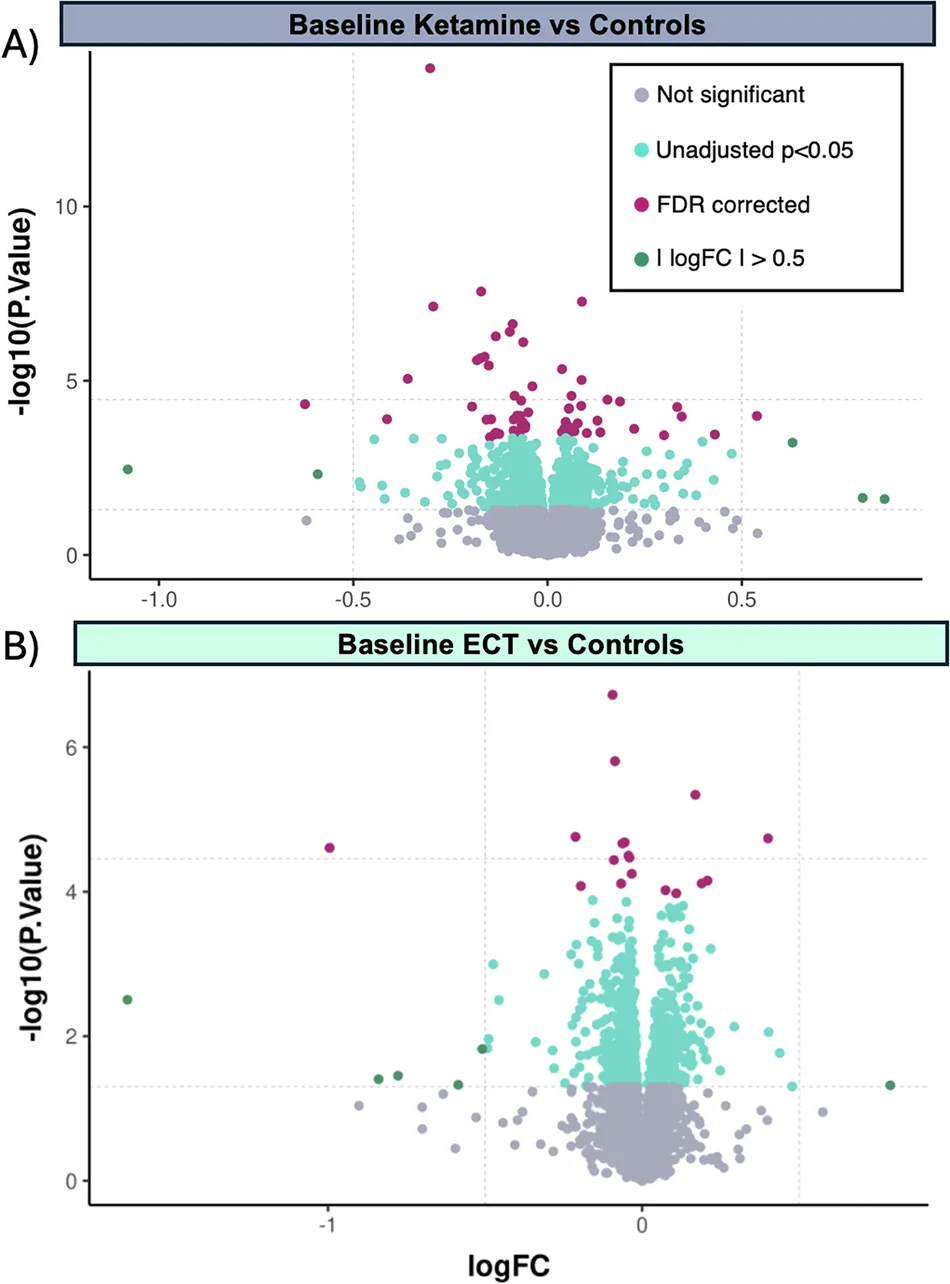
Baseline differential gene expression between controls and patients with TRD prior to receiving treatment. Volcano plot for baseline differential gene expression comparing HC and **A** patients receiving ketamine and **B** patients receiving ECT.

No significant transcriptional changes were detected in the ketamine group after multiple comparison correction (Table 2). Trending (uncorrected p < 0.05, |logFC| >0.5) interactions for time*remission status were observed in 3 genes, and an additional 3 genes for time*diagnosis when compared to controls. Since none survived FDR, gene ontology analysis was not performed. Baseline FDR-corrected analysis identified 1 upregulated and 4 downregulated genes in remitters vs. non-remitters. However, only one of these genes (IGKV1-9) had a significant logFC (p = 0.000025, logFC = −0.51). Functional classification suggests this gene is involved in molecular functions related to immune response and immunoglobulin production. When comparing baseline gene expression of all patients receiving ketamine to controls, 64 genes were differentially expressed after FDR (Fig. 2). However when applying the logFC threshold, only 1 upregulated gene (RPL36AP37, FDR adjusted p = 0.02, logFC = 0.53) and one down regulated gene (RNF144B, FDR adjusted p = 0.016, logFC = −0.62) remained; functional annotation revealed that RPL36AP37 is a ribosomal pseudogene with no known protein-coding function, while RNF144B encodes an E3 ubiquitin ligase implicated in the regulation of apoptosis and inflammatory signaling pathways.

#### ECT DGE analysis

Two genes showed significant change after FDR correction following ECT when comparing baseline to post-index treatment. RPL26P5 was downregulated post-index when compared to baseline (FDR adjusted p = 0.008, logFC = −0.15) and EIF1AY was upregulated post-index compared to baseline (FDR-adjusted p = 0.008, logFC = 0.23), though neither had a logFC greater than 0.5 or smaller than −0.5. After FDR correction, no significant interactions were observed between the change to post-index with treatment response or diagnosis when compared to controls. Significant changes were also absent from baseline to follow-up. At baseline, no significant DGE was observed between responders and non-responders after FDR correction. When comparing pre-treatment patients receiving ECT and controls, 18 genes were differentially expressed (Fig. 2), however, only 1 gene had a substantial logFC (HLA-DQA1, FDR adjusted p = 0.02, logFC = −0.99); gene ontology analysis identified its role in antigen processing and presentation via MHC class II.

### Network analysis

#### Ketamine network analysis

Network analysis revealed no significant associations with module eigengenes (ME) for any analyses after FDR correction. Trending associations (unadjusted p < 0.05) can be found in Supplemental Table 4.

#### ECT network analysis

A significant interaction between time and diagnosis was observed in 8 modules (ME3, ME2, ME10, ME11, ME21, ME16) after correcting for FDR (Supplemental Table 4). However, none achieved sufficient logFC thresholds and post-hoc analyses revealed no changes reaching significance in ECT or controls (Supplemental Fig. 14). At baseline, 12 eigengenes significantly differed between ECT patients and controls (ME14, ME2, ME23, ME10, ME13, ME3, ME15, ME9, ME7, ME21, ME11, ME1; Supplemental Table 4, Supplemental Fig. 16) but none had sufficient logFC.

## Discussion

We investigated whole blood transcriptomics as a possible biomarker for rapid-acting antidepressant treatments, including sub-anesthetic ketamine and ECT. Despite the highly significant antidepressant effects observed in these two different TRD treatment cohorts, we failed to detect significant longitudinal changes in gene expression over time or in association with clinical response after employing rigorous and necessary multiple comparison correction methods. Notwithstanding, our results provide some preliminary evidence that cross-sectional pre-treatment transcriptional profiles differ with TRD diagnosis and future treatment response.

### Treatment-related changes in gene expression

Only a few prior studies have examined peripheral blood gene expression following antidepressant treatment, and findings have been inconsistent with limited overlap across cohorts [32–37, 44–46]. Even relatively large SSRI trials have reported minimal longitudinal effects, suggesting that antidepressant response may exert subtle, distributed transcriptional changes that are difficult to detect [37]. We hypothesized that rapid-acting treatments such as ketamine and ECT would provide a more favorable context for capturing such effects, given their robust clinical impact within days. However, despite observing strong clinical improvements and changes in brain structure [55, 56] and function [57–60] previously reported in overlapping samples, we were unable to detect significant or clearly clinically meaningful longitudinal gene expression changes following either intervention [61, 62].

Our findings together with the limited prior data available suggest that changes in gene expression induced by ketamine and ECT are subtle, and potentially heterogeneous, requiring much larger samples to capture. Another possibility is that our failure to observe significant longitudinal changes in gene expression was due to the sampling of peripheral blood. Although there is some overlap between gene expression in blood and brain—particularly for genes implicated in depression [28]— the correspondence is variable, with stronger correlations observed for immune-related genes than for other functional categories [26]. This observation is notable but should be interpreted in light of the fact that blood is a primary site of immune activity, and thus immune-related transcripts are often prominent in peripheral gene-expression studies. Still, most transcriptional alterations associated with antidepressant response are expected to occur within brain tissue, which remains inaccessible for in vivo experiments [26]. That is, while postmortem brain studies have advanced knowledge of brain-specific gene expression, their cross-sectional nature limits their utility in capturing dynamic treatment effects [63]. Emerging models such as brain organoids derived from induced pluripotent stem cells may eventually provide complementary insights, although these systems remain limited in their ability to capture the architectural, vascular, and immune complexity of the human brain [64–66]. These considerations underscore the importance of integrating multiple experimental approaches—spanning peripheral markers, neuroimaging, postmortem studies, and organoid models—to more comprehensively characterize the systemic and dynamic mechanistic effects of rapid-acting antidepressants.

### Depression-related alterations in gene expression

When looking cross-sectionally, we observed a small number of differentially expressed genes distinguishing ketamine remitters from non-remitters, as well as patients receiving ketamine or ECT compared to healthy controls, though only one gene in each comparison showed a substantial effect size after FDR and logFC thresholds. Although ontology annotations for these isolated genes point toward inflammatory and immune signaling pathways—a tempting interpretation given prior work—genes rarely act in isolation, and we did not observe convergent evidence at the network level. Thus, we caution placing strong interpretive weight on these isolated findings. It is potentially more likely that they represent fragments of a broader biological signal that is not well captured at the single-gene level. Still, the detection of even a handful of differences suggests there may be weak but real transcriptional effects that become apparent at a cross-sectional level, particularly in TRD where the cumulative burden of illness could leave a detectable imprint in peripheral blood gene expression. Existing studies examining DGE in psychiatric disorders have produced mixed results. For MDD, some studies have identified diagnostic differences in blood-based gene expression [67], while others have reported no significant associations [68]. Similarly, findings in bipolar disorder (BD) have been inconsistent. Some studies have reported significant DGE in BD [69, 70] but it has been suggested that gene expression patterns observed in BD patients may be primarily due to lithium use [71]. While these inconsistencies may be partly attributable to differences in study design and sample size, they are also likely driven by the inherent heterogeneity of mood disorders, including variability in symptom profiles, treatment histories, and underlying neurobiology. These conflicting findings underscore the notion that altered peripheral gene expression effects in psychiatric disorders are likely modest, highly context-dependent, and only visible in much larger datasets. It is possible that diagnostic group effects may have been biased by including a treatment-resistant cohort with severe, chronic illness. This may also explain the larger control–ECT differences, as ECT patients had greater symptom severity and comorbidity than the ketamine group at baseline (Supplemental Table 3). Furthermore, it is possible that we were able to detect some cross-sectional treatment-related effects because individuals who do not respond to ketamine or ECT may have a more unique form of TRD and carry a distinct biological signature; one that is more pronounced, and thus more likely to be reflected in peripheral blood. Alternatively, this profile may reflect the cumulative effects of a prolonged treatment history, with multiple medications potentially leaving a lasting biological imprint. In contrast, responders and remitters may exhibit more transient or subtle molecular changes or perhaps reflect a more plastic or adaptive transcriptional profile. Thus, illness severity, chronicity, and treatment resistance may indeed be reflected in peripheral gene expression; however, these effects require large, well-powered samples to detect reliably.

### Network analysis findings

While we observed some significant time-by-diagnosis and time-by-remission interactions after clustering genes into co-expression networks using WGCNA, results were modest (|logFC| <0.05), and the functional classification of genes within significant modules were somewhat uninformative. WGCNA was chosen for its strength in reducing dimensionality, which is important for addressing Type I error and because genes rarely function in isolation. Modules were constructed using data from all time points across all groups (ECT, ketamine, and controls) to capture patterns of gene co-expression across the full sample. Understanding how genes cluster and act together is likely more informative than analyzing individual genes alone. One module, ME21, emerged in several analyses. At baseline, ME21 expression was significantly higher in controls compared to the ECT group (passing FDR correction). In the ketamine group, no results survived FDR correction, but trending results showed non-remitters had higher baseline ME21 expression than remitters. A time-by-remission interaction showed modest ME21 increases in remitters, with no change in non-remitters. Functionally, ME21 was enriched for genes related to immune response and immunoglobulin production, which aligns with hypotheses about immune dysregulation in depression and potential anti-inflammatory effects of ECT and ketamine. However, further inspection revealed that the gene ontology signal was primarily driven by a single gene, IGKV1-9, which was also identified in our differential gene expression analysis. Ideally, biologically meaningful pathway enrichment would be supported by coordinated changes across multiple genes. Given that this is just one gene and the direction of effects was inconsistent, these findings are not sufficient to interpret within an immune-related framework.

### Limitations

As discussed above, our modest sample size (N = 97 participants with TRD, N = 35 unaffected controls) may have limited our ability to detect subtle or distributed transcriptional changes, particularly after correction for multiple comparisons. While a subset of controls underwent repeated sampling, a placebo or sham treatment group was not included because of the naturalistic design of the parent study and ethical issues in treating healthy controls with ketamine or ECT. Nevertheless, healthy controls did not exhibit significant longitudinal changes in gene expression, suggesting that nonspecific or placebo effects are unlikely to account for our findings. Moreover, in this same cohort, we have previously reported robust treatment-related changes in brain structure [55, 56] and functional networks [57–60], and in the ECT sample, we observed significant changes in cytokine measures [72]. Taken together, these findings support the presence of treatment-specific effects, although the absence of a formal placebo condition remains a limitation for fully isolating treatment-specific molecular changes. It is also worth taking into consideration that the control group was significantly younger than the TRD group, which may have contributed to baseline differences in gene expression and represents a potential confound. Though the ECT group exhibited somewhat lower response rates than those reported in some samples and meta-analyses, this difference may reflect sample composition (e.g., older age, presence of psychotic depression), treatment protocols (primarily right unilateral stimulation), or broader clinical heterogeneity, all of which should be considered when interpreting our findings. Still, the clinical responses we observed are consistent with the well-established efficacy of ketamine and ECT [12, 13, 73], supporting the interpretation that these changes were likely attributable to active treatment.

Regarding timing of blood collection, blood was collected 24 h post-treatment, when antidepressant effects are typically strongest for ketamine and ECT. However, some rapid and transient transcriptional changes may have occurred within hours of treatment and were thus not captured. That said, if ketamine or ECT exert meaningful downstream effects on immune or inflammatory pathways—as suggested by prior work in mostly modest sample sizes—one might expect such signals to persist for at least 24 h and remain detectable. The absence of robust effects 24 h post-treatment raises further questions about the magnitude or consistency of these peripheral molecular signatures.

## Conclusion

Our findings may benefit future studies investigating the molecular mechanisms of antidepressant response. The lack of robust longitudinal changes in peripheral gene expression suggests that RNA-seq may have limited immediate translational utility, and alternative approaches—such as DNA methylation, proteomics, or multimodal integration—may prove more informative. While ketamine and ECT produced clear clinical benefits in TRD, we failed to detect clear and consistent transcriptional signatures in peripheral blood relating to treatment. Modest cross-sectional differences hint at underlying biological distinctions, but larger discovery studies with greater statistical power and refined phenotyping will be critical for identifying reliable biomarkers.

## Supplementary information

Supplemental Material

## Data Availability

The gene expression data generated is available through the NCBI Gene Expression Omnibus (GEO) accession number GSE341751. Additional metadata for this study are available through the NIMH Data Archive (NDA Collection #2844).
