# Supplementary material for: Transcriptional profiling of antidepressant ketamine and electroconvulsive therapy treatment
Source: Transl Psychiatry. 2026 Aug 5;16:603. doi: 10.1038/s41398-026-04196-y (PMC13724684; doi:10.1038/s41398-026-04196-y)
Supplement: Supplementary file 1 [file 41398_2026_4196_MOESM1_ESM.pdf]

Demographics Tables

Supplemental Table 1. Mean age and sex distributions of participants with available plasma samples at each time point during ECT or ketamine treatment

	N	Age	Percent Female
Ketamine			
Baseline	60	39.5±11.2	46.7%
24 hrs post 1st infusion	39	38.7±10.9	35.9%
24 hrs post 4th infusion	52	39.3±10.7	48.1%
5-week follow up	37	40.1±10.2	43.2%
ECT			
Baseline	37	41.6±16.0	67.6%
<1 week after the ECT index	32	40±16.0	62.5%
3-month follow up	20	37.7±13.3	60%

Supplemental Table 2. Race and education of study participants

**Education (%)**				**Race (%)**			
	KET	ECT	HC		KET	ECT	HC
10th grade	1.54%	0%	0%	White, Non-hispanic	70.77%	73.17%	25.64%
High School Graduate	3.08%	2.44%	2.56%	White, Hispanic/latino	7.69%	2.44%	17.95%
Some College	27.69%	26.83%	20.51%	Asian	10.77%	7.32%	17.95%
Associates Degree	4.61%	12.19%	2.56%	Black	1.54%	4.88%	17.95%
Bachelor's Degree	33.85%	29.27%	46.15%	More than one race	3.08%	4.88%	7.69%
Masters Degree	20%	14.63%	17.95%	Other	4.61%	7.32%	12.82%
Professional Degree	6.15%	14.63%	7.69%	Not reported	1.54%	0%	0%
Doctoral Degree	3.08%	0%	2.56%				

Supplemental Table 3. Concurrent medication and psychiatric comorbidities. All participants were diagnosed with treatment resistant depression and were experiencing a major depressive episode at the time of the study. Other diagnoses were defined using a structured clinical interview for DSM (SCID).

	Subcategory	KET (%)	ECT (%)
**Medications**	SSRI	30.77	24.39
	SNRI	30.77	26.82
	Other Antidepressants	52.31	43.90
	Lithium	1.53	12.19
	Benzodiazepines*	29.23	31.70
	Anticonvulsants	24.61	39.02
	Atypical Antipsychotics	20.00	36.58
	Stimulants	24.61	4.88
	No Current Psychotropic Medication	20.00	0.00
**Other Diagnoses**	MDD with Bipolar Features	3.39	24.39
	PTSD	22.03	46.34
	Anxiety	62.71	65.85
	History of Cannabis Dependence	10.17	14.63
	History of Alcohol Dependence	27.12	41.46
	History of Nicotine Dependence	9.23	17.07

*Patients were requested to withhold benzodiazepines >24 hrs before treatment sessions and blood draws

Supplemental Table 4. WGCNA network analysis findings

Module	Model	logFC	Raw p-value	Adjusted p-value
Ketamine				
ME21	time*remission	-0.04	0.03	0.7
ME21	baseline remission	-0.04	0.009	0.22
ME12	baseline remission	0.03	0.02	0.22
ME15	baseline remission	0.03	0.03	0.22
ME10	baseline remission	0.02	0.04	0.22
ME22	Baseline diagnosis	-0.02	0.03	0.6
ECT				
ME3	time*diagnosis	0.012	0.0002	0.004
ME1	time*diagnosis	-0.003	0.0003	0.004
ME14	time*diagnosis	0.005	0.001	0.005
ME2	time*diagnosis	-0.002	0.001	0.005
ME10	time*diagnosis	-0.026	0.004	0.018
ME11	time*diagnosis	0.016	0.006	0.023
ME21	time*diagnosis	-0.007	0.013	0.040
ME16	time*diagnosis	-0.016	0.013	0.040
ME14	baseline diagnosis	-0.06	0.00003	0.001
ME2	baseline diagnosis	0.06	0.0001	0.002
ME23	baseline diagnosis	0.05	0.001	0.010
ME10	baseline diagnosis	0.06	0.004	0.023
ME13	baseline diagnosis	-0.04	0.007	0.033
ME3	baseline diagnosis	-0.06	0.008	0.033
ME15	baseline diagnosis	0.04	0.010	0.033
ME9	baseline diagnosis	0.05	0.011	0.033
ME7	baseline diagnosis	-0.04	0.013	0.033
ME21	baseline diagnosis	0.04	0.014	0.033
ME11	baseline diagnosis	0.03	0.016	0.036

ME1	baseline diagnosis	-0.04	0.018	0.036
ME11	Baseline responder	0.03	0.009	0.2

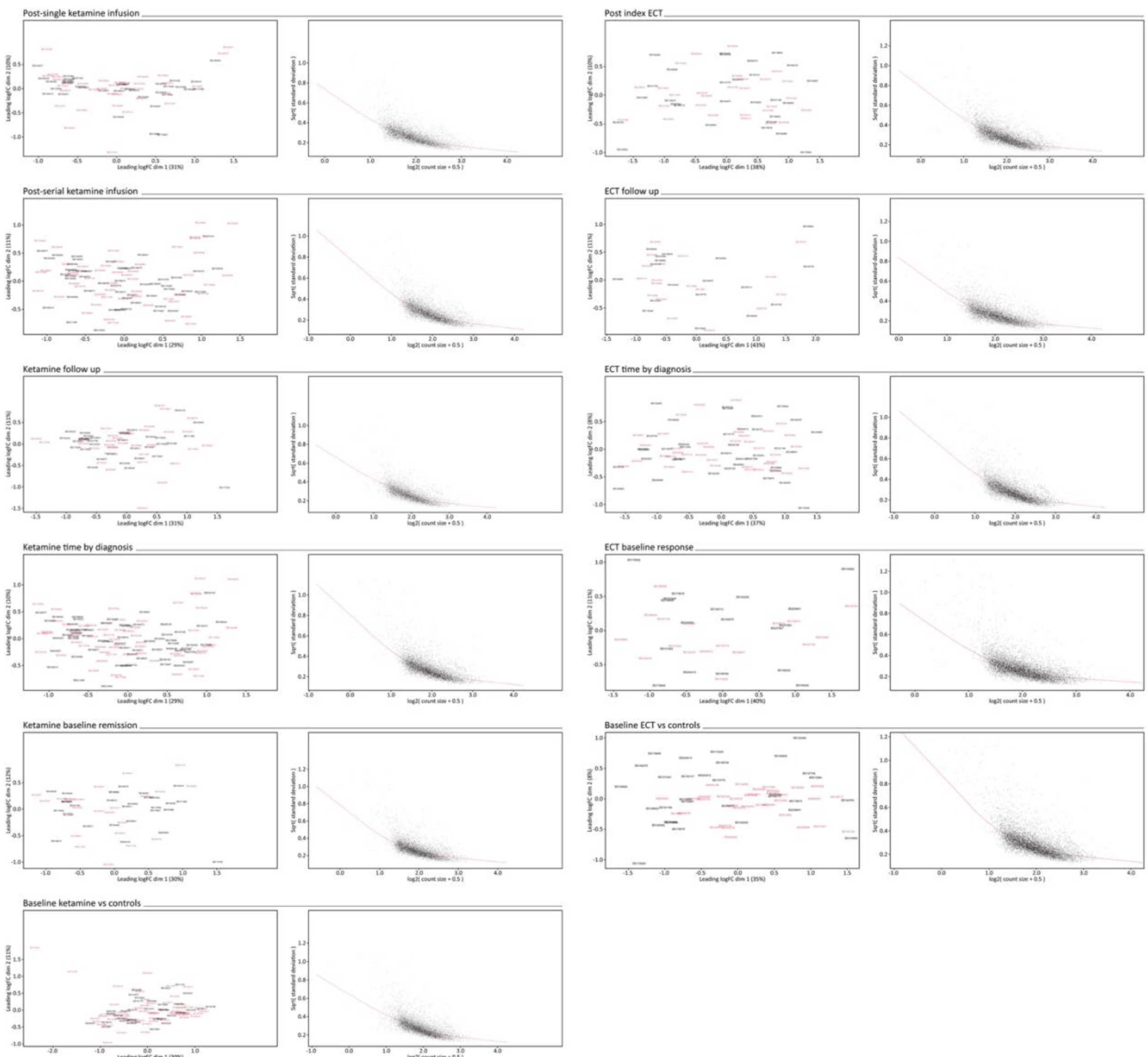

Supplemental Figure 1. Multidimensional scaling (MDS) plot and limma-voom mean-variance trend J-plots for each analysis.

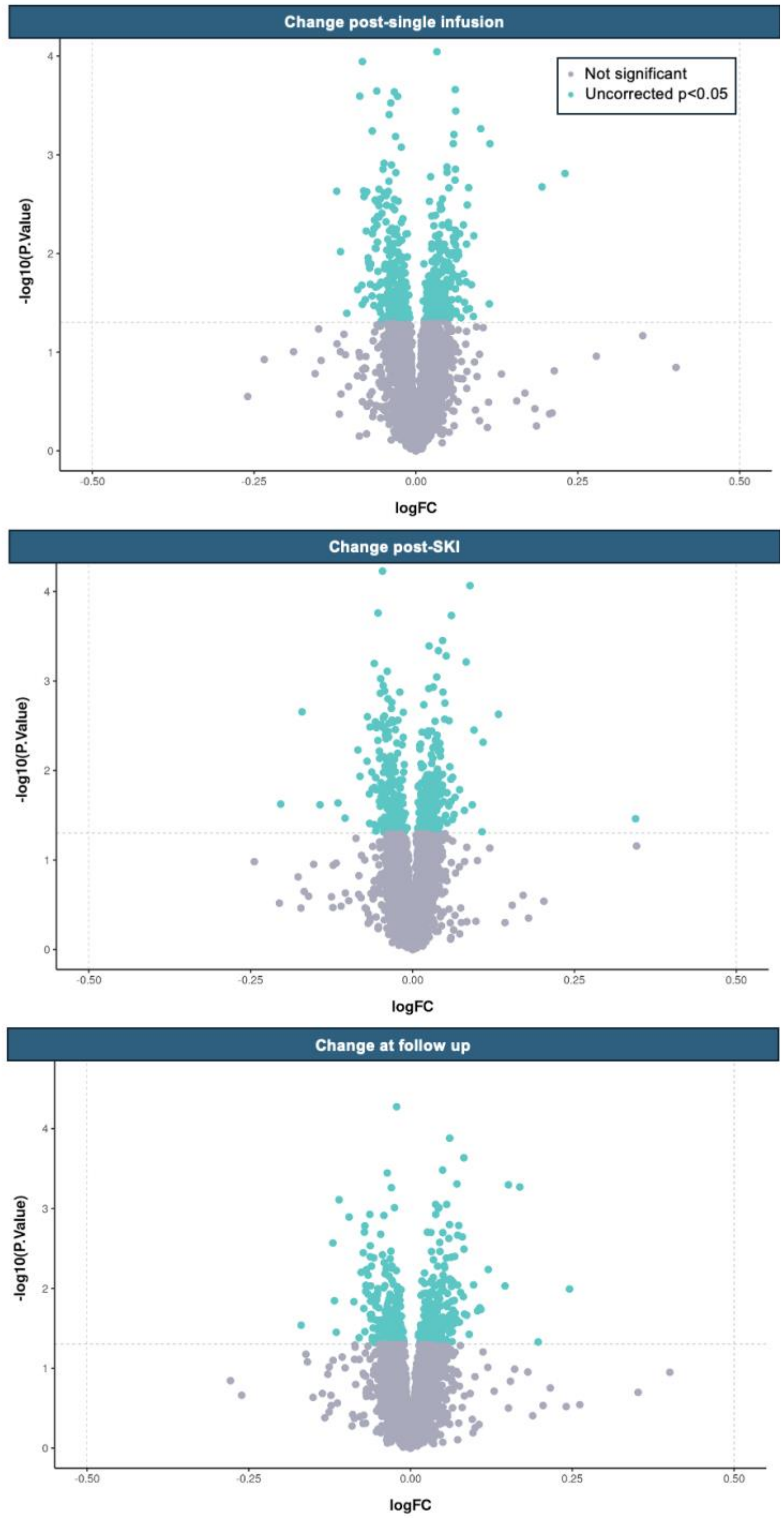

Supplemental Figure 2. Volcano plots for pairwise DGE analysis in the ketamine sample. Comparisons are base with baseline and each timepoint.

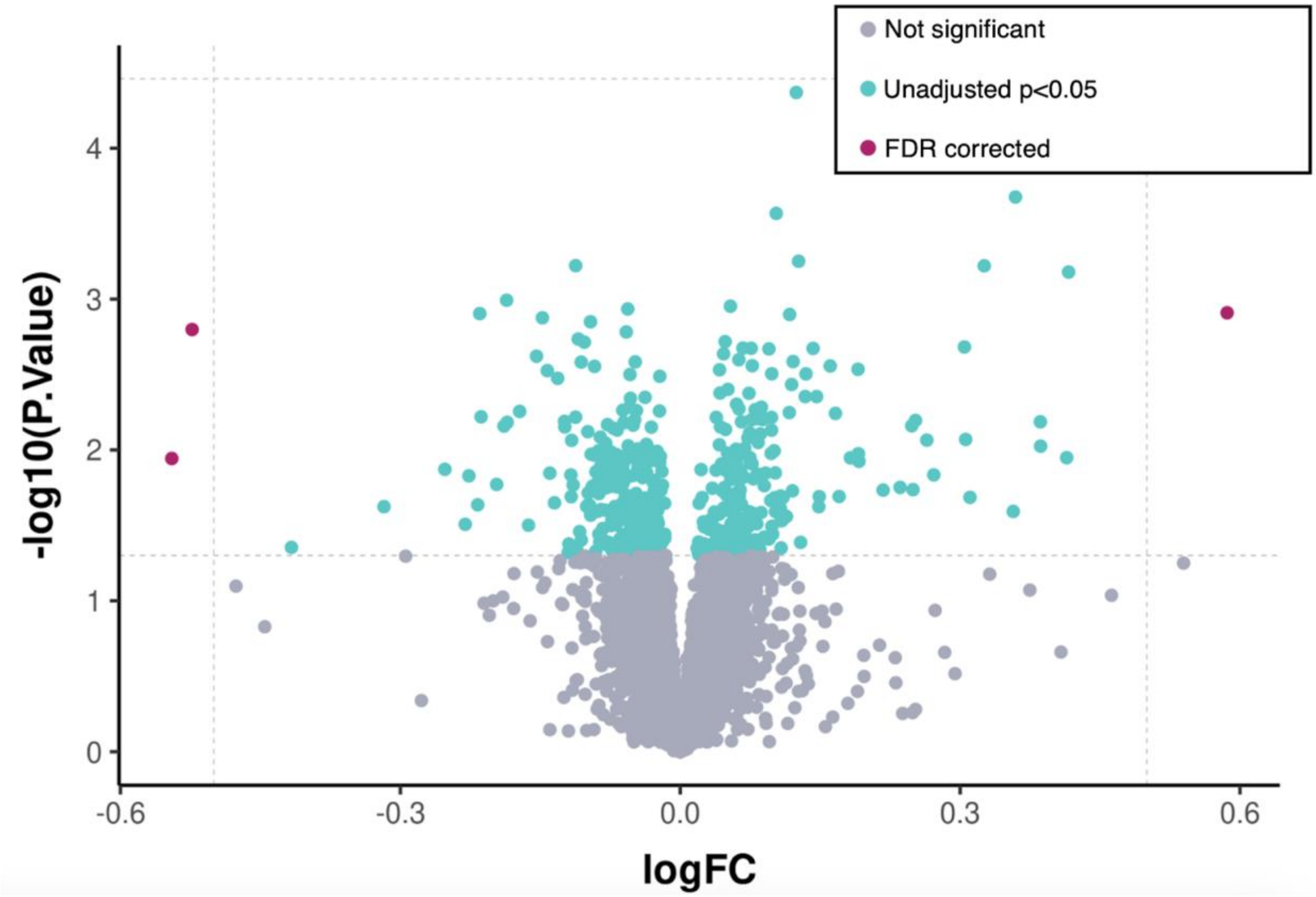

Supplemental Figure 3.1. Ketamine sample volcano plot for results comparing time*remission status when comparing from baseline to SKI.

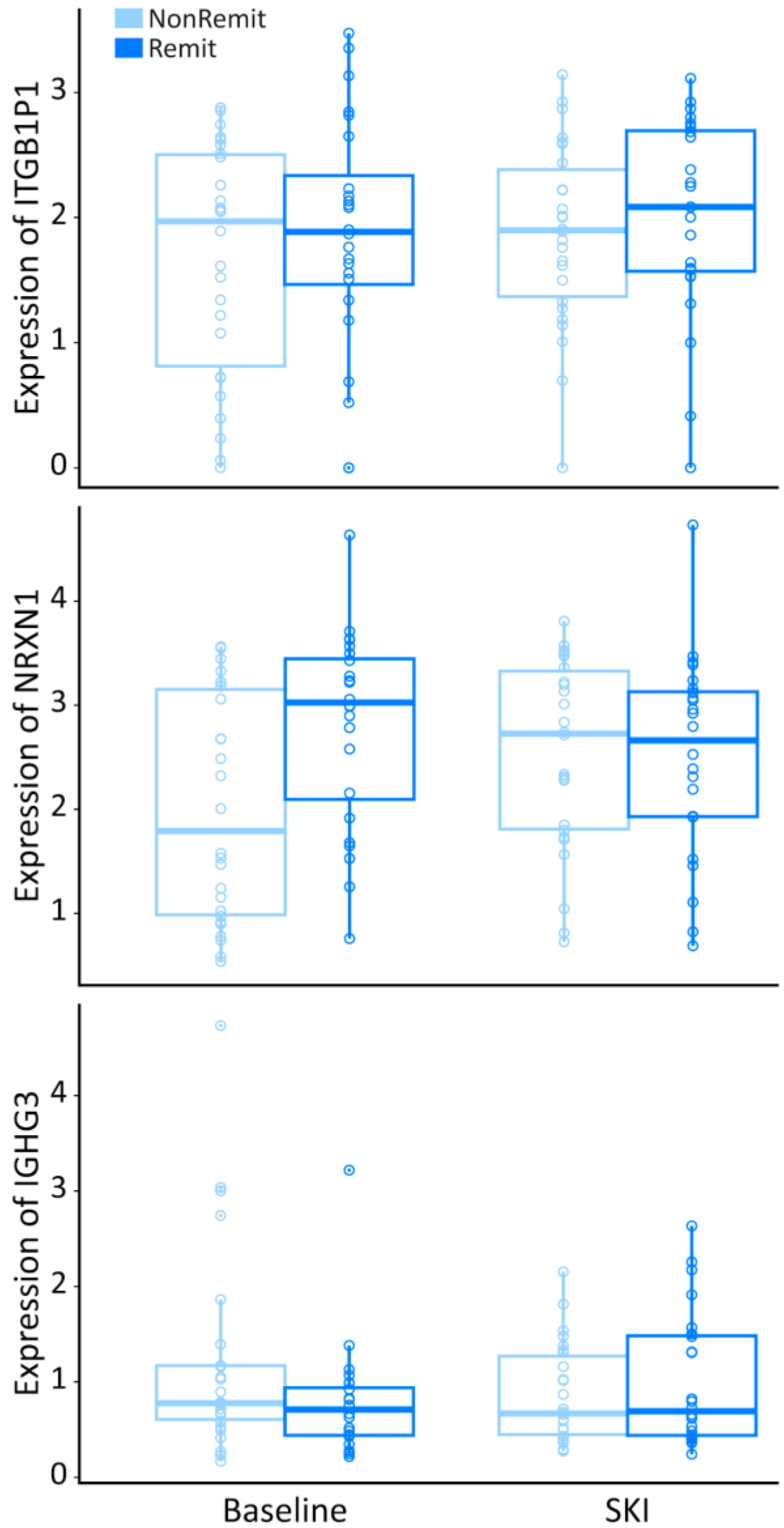

Supplemental Figure 3.2. Boxplots showing differences between ketamine patients defined as remitters and non-remitters at baseline and following serial ketamine for trending genes extracted from the time*remission interaction.

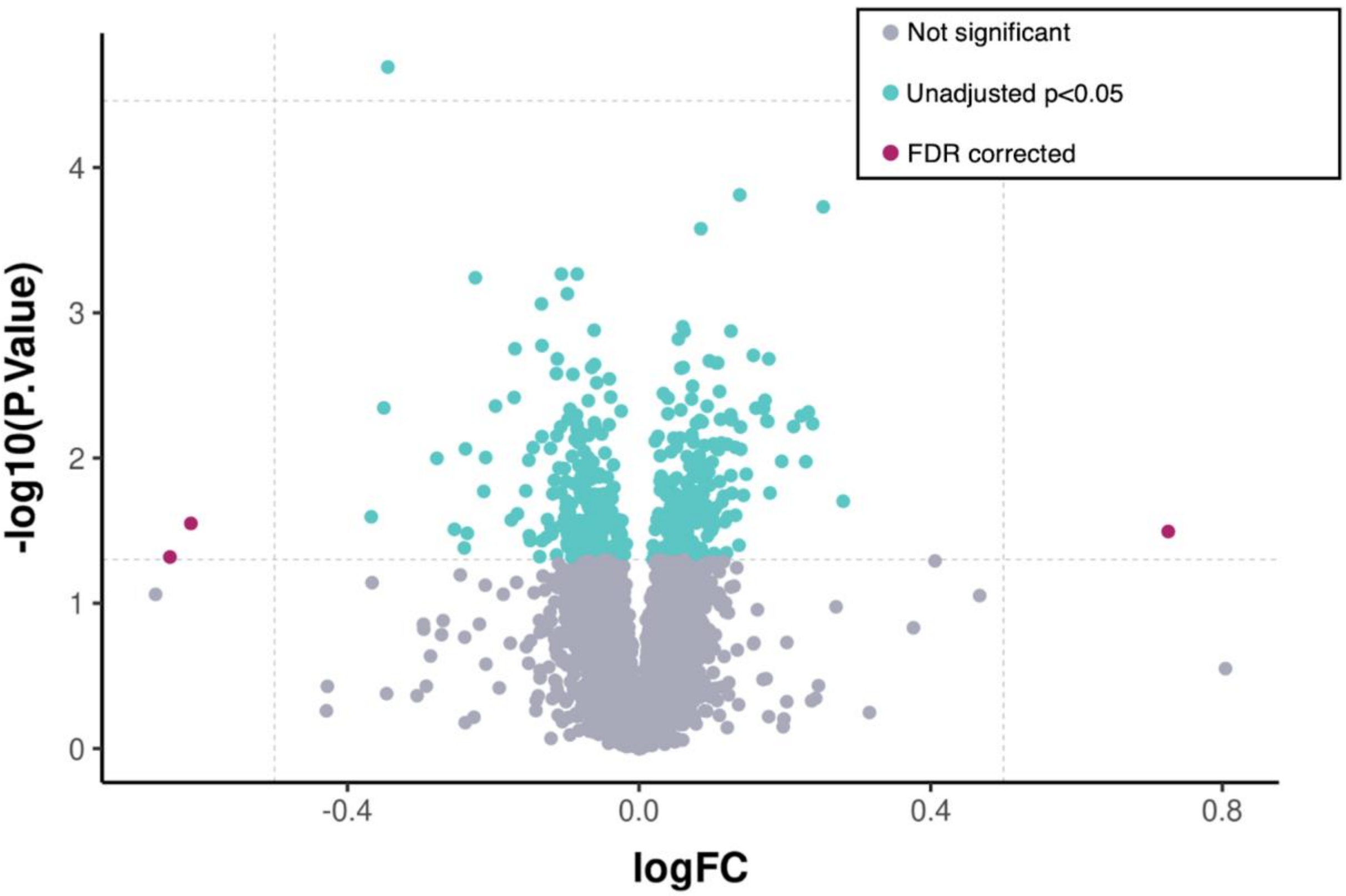

Supplemental Figure 4.1. Ketamine sample volcano plot for results comparing time*diagnosis.

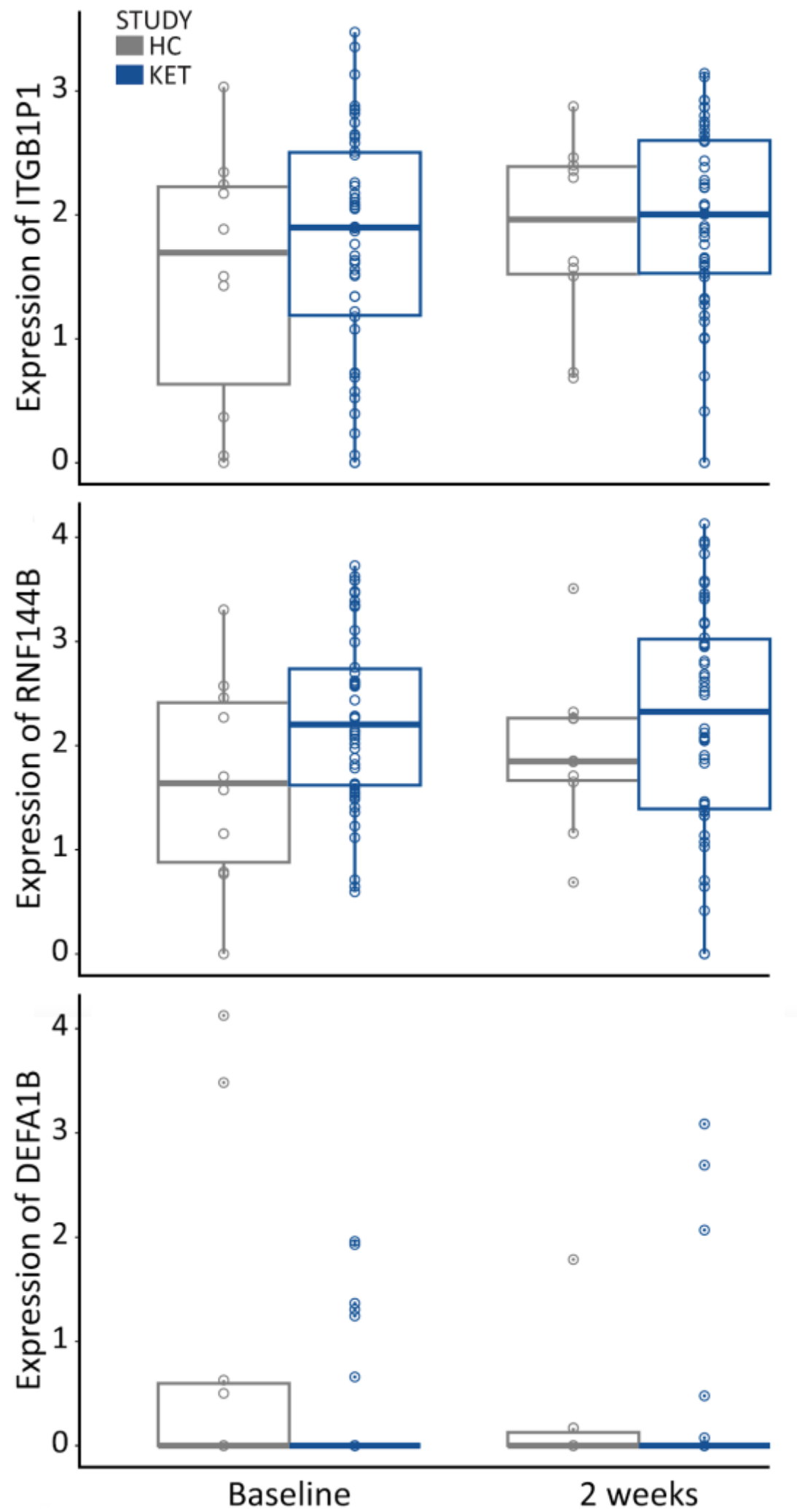

Supplemental Figure 4.2. Boxplots showing differences between ketamine patients and controls at baseline and after 2-weeks for controls and serial ketamine for patients for genes surviving FDR correction from the significant genes for time*diagnosis analysis.

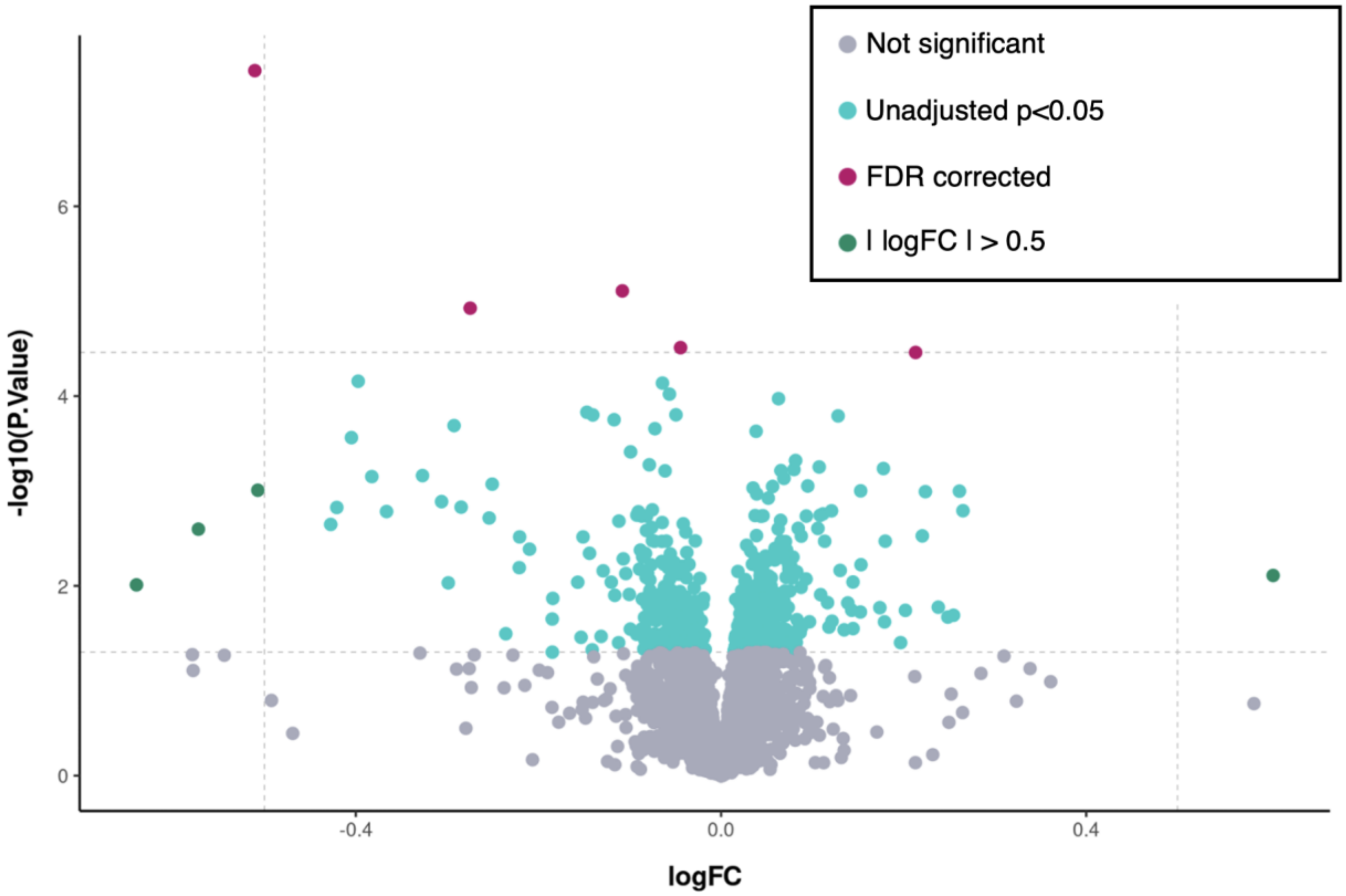

Supplemental Figure 5. Ketamine sample volcano plot for results comparing remitters versus not remitters at baseline.

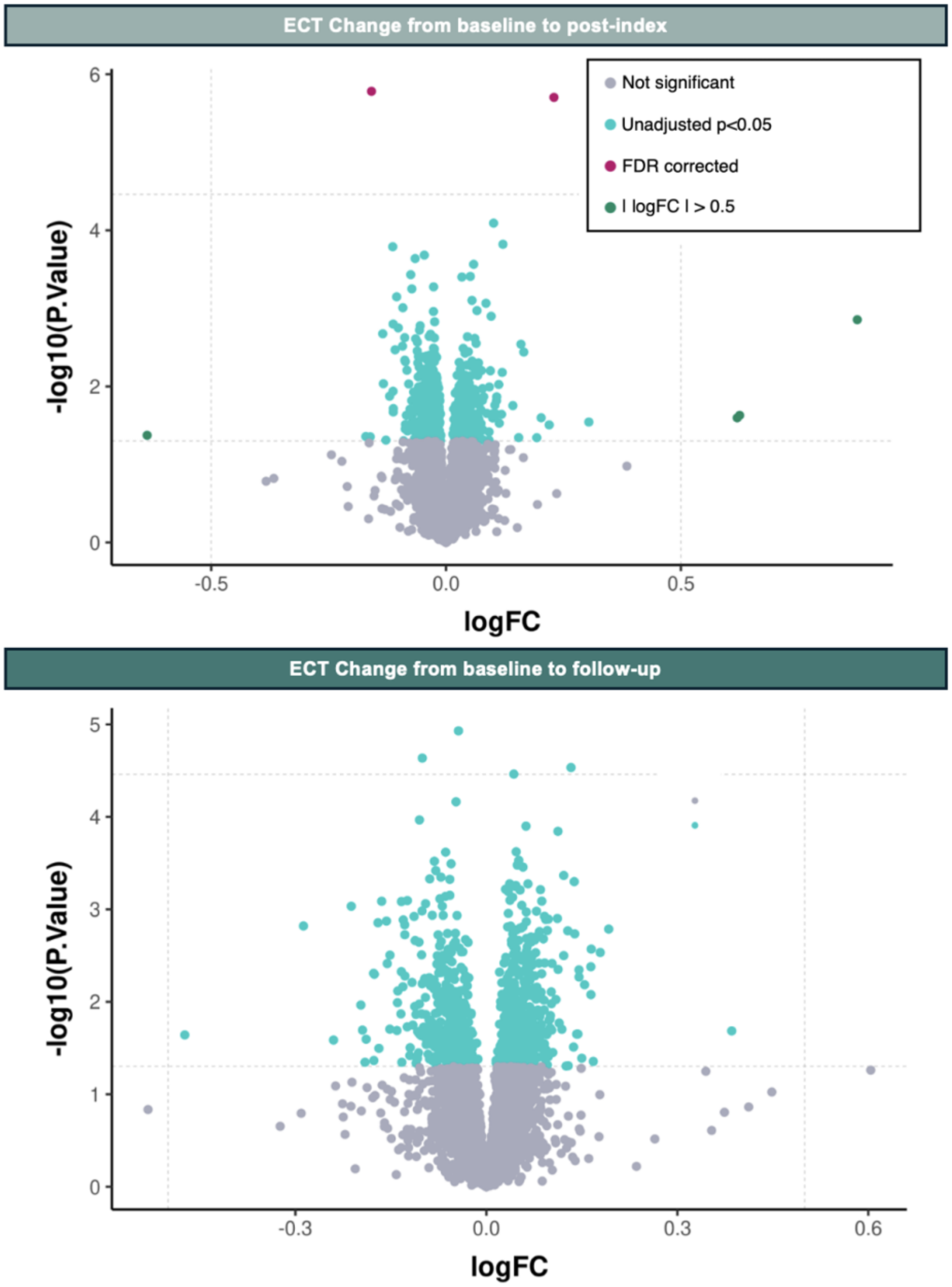

Supplemental Figure 6. ECT sample volcano plot for results comparing longitudinal changes from baseline to post-index.

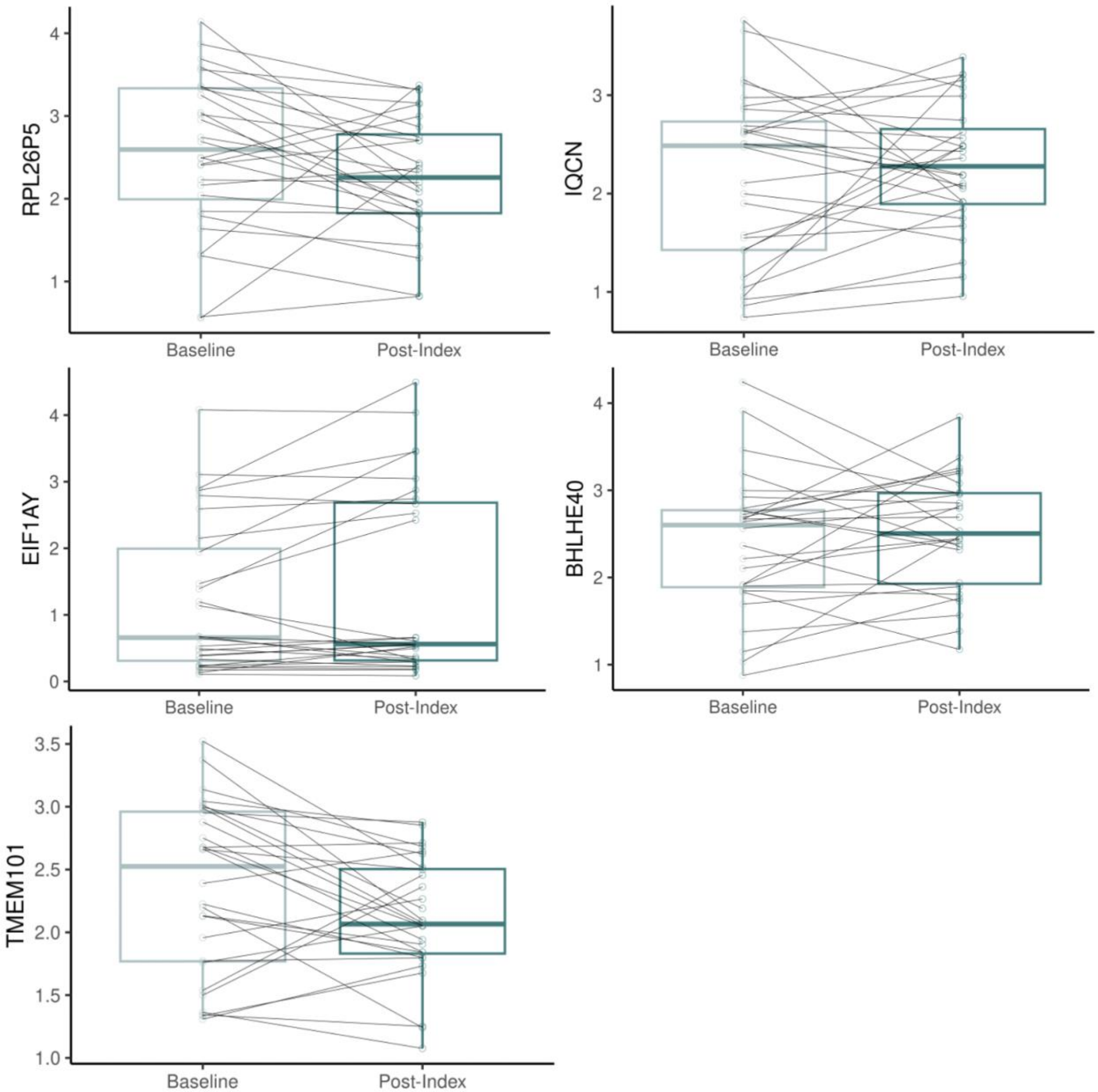

Supplemental Figure 7. Boxplots showing genes with trending changes in the ECT sample from baseline to post-index.

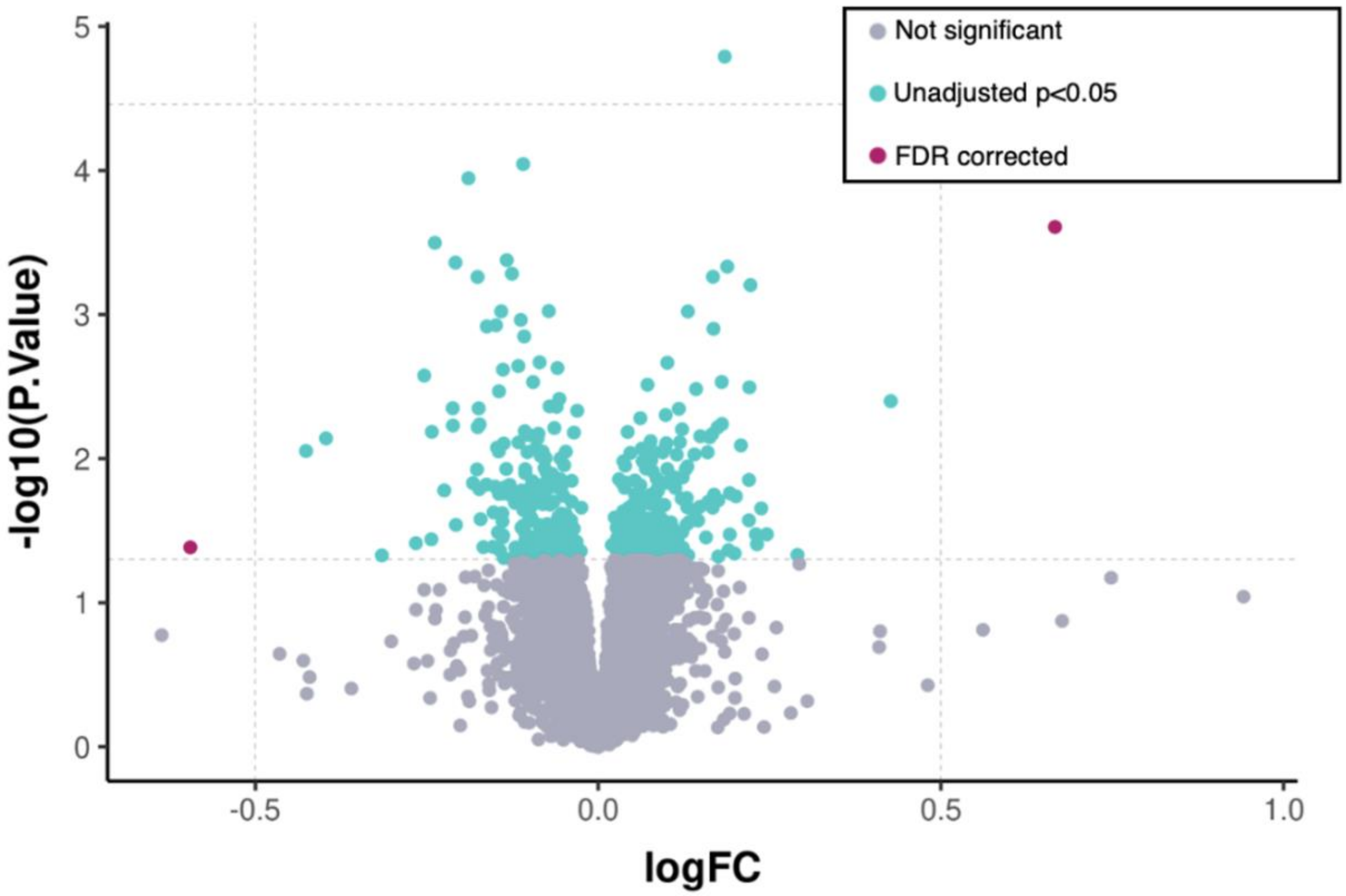

Supplemental Figure 8. ECT sample volcano plot for results investigating time*diagnosis.

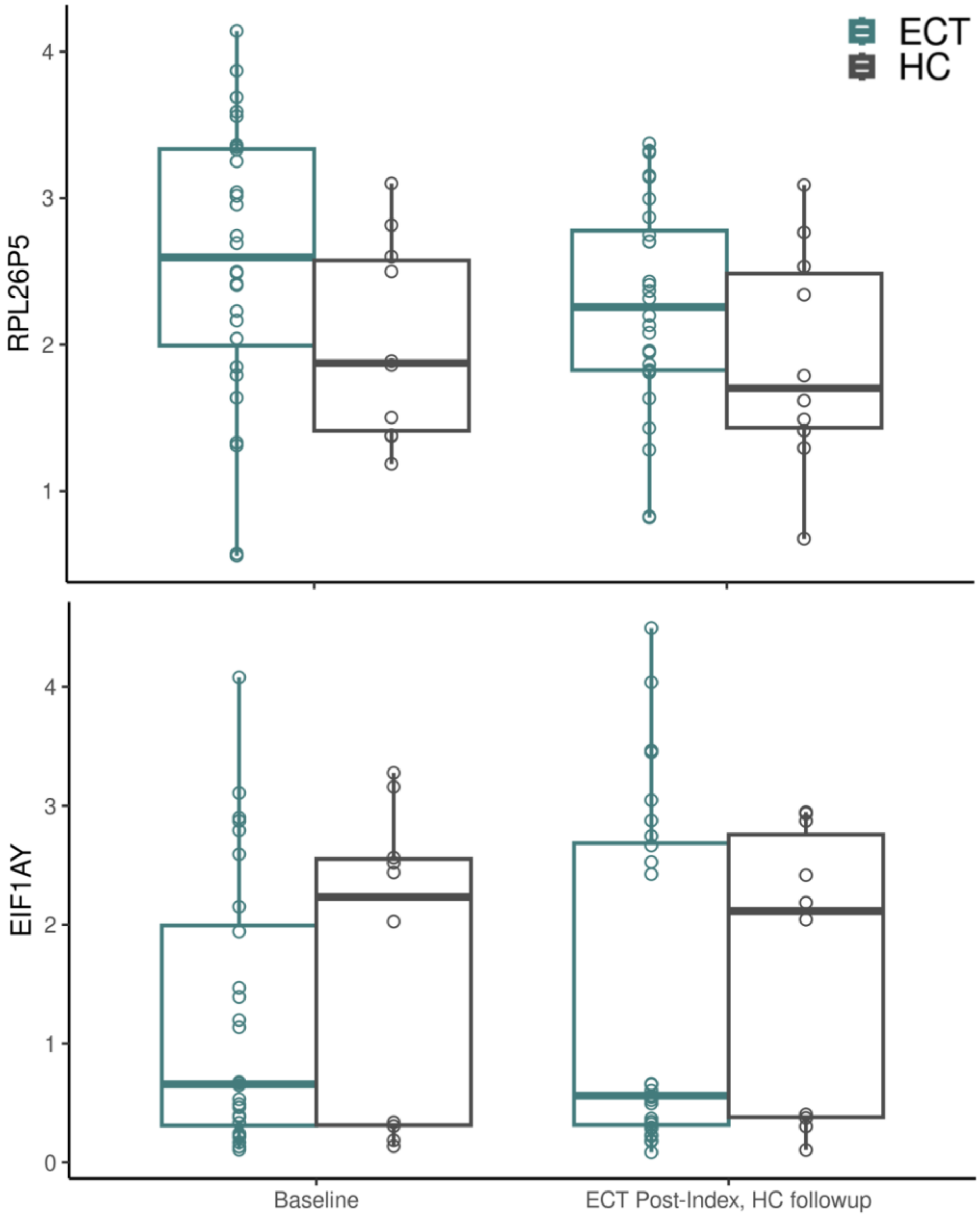

Supplemental Figure 9. Boxplots showing longitudinal changes for ECT (post-index) and controls (follow up) for genes that were trending in significance for a time*diagnosis interaction.

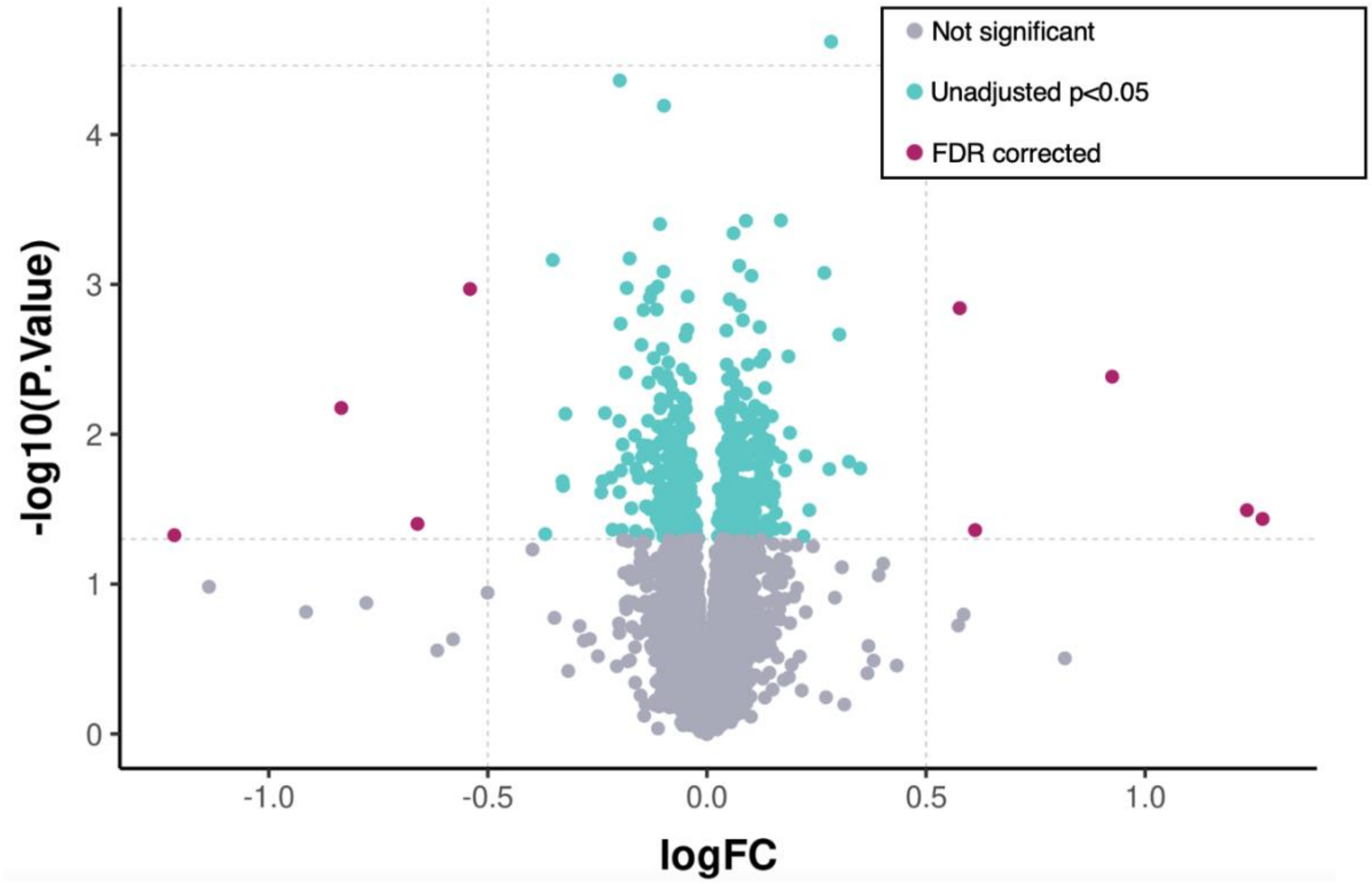

Supplemental Figure 10. ECT sample volcano plot for results comparing responders and non-responders at baseline.

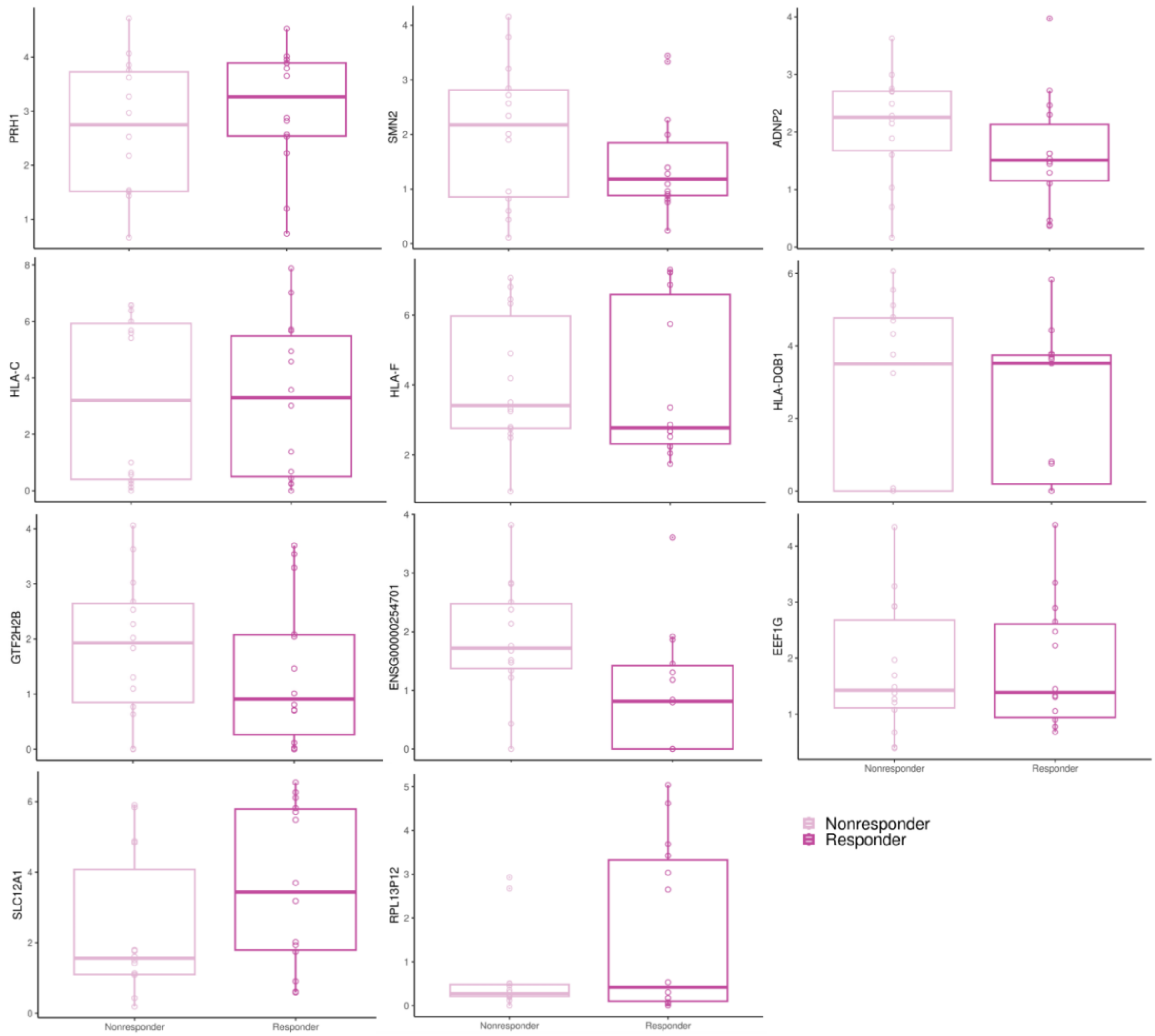

Supplemental Figure 11. Boxplots of trending genes differentially expressed when comparing ECT responders and non-responders at baseline.

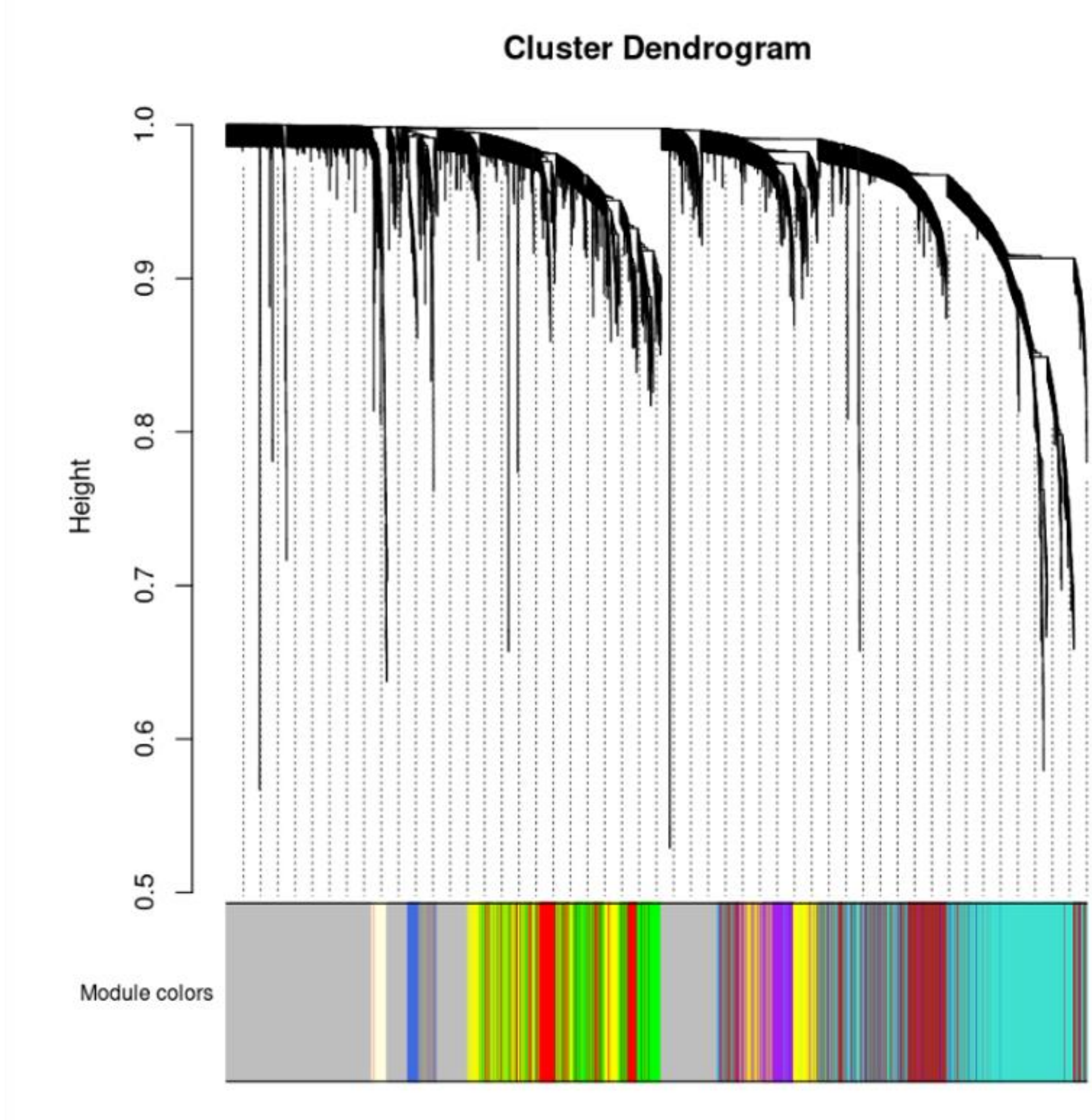

Supplementary Figure 12. Cluster dendrogram for WGCNA created using data across time points and groups (ECT, Ketamine, controls).

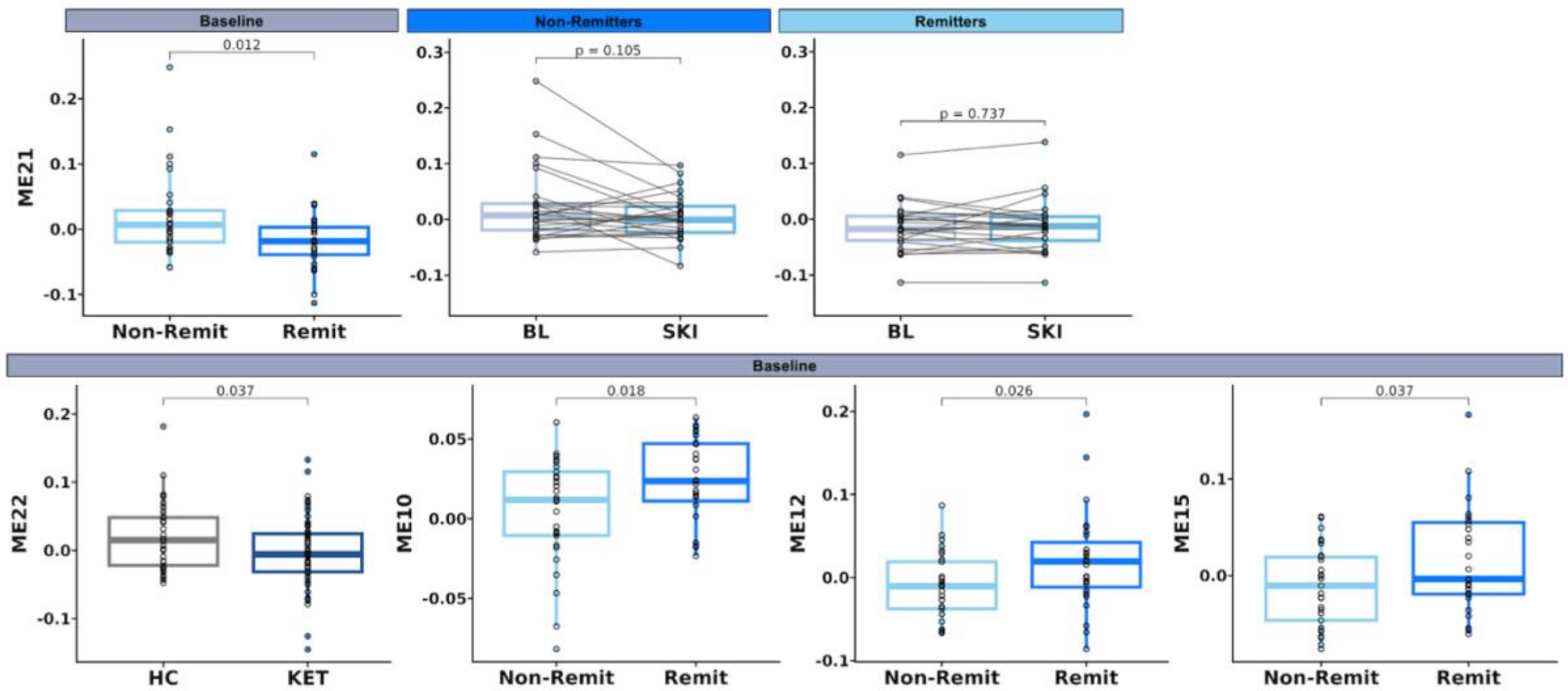

Figure 13. Trending eigengene results within the ketamine sample. A time-by-remission interaction was observed for ME21 (p<0.05), with remitters showing a modest post-treatment increase. At baseline, ME21, ME15, ME12, and ME10 differed by remission status, and ME22 differed by diagnosis. All p-values are unadjusted.

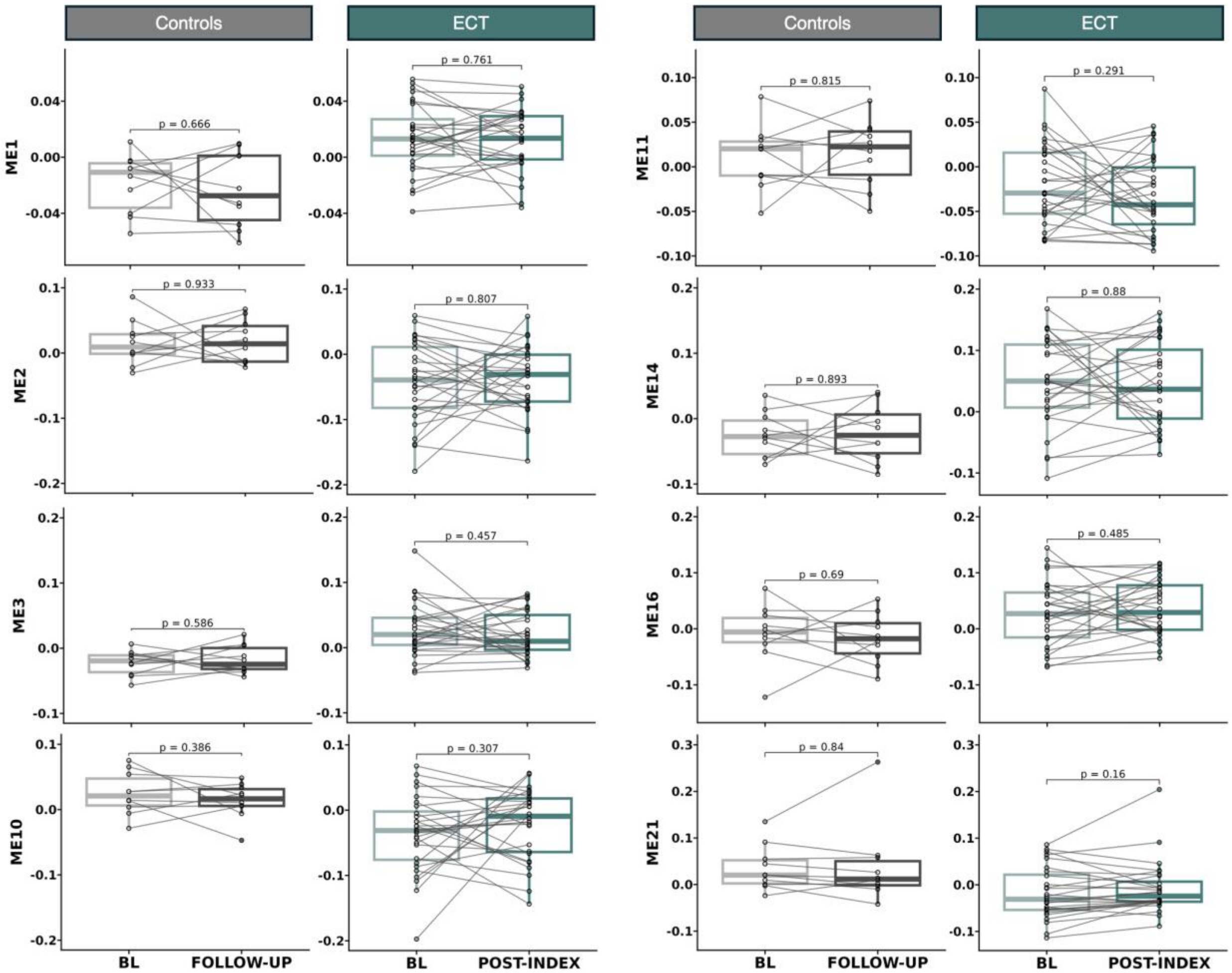

Supplemental Figure 14. Boxplots for significant eigengene findings for results from the time*diagnosis analyses for ECT.

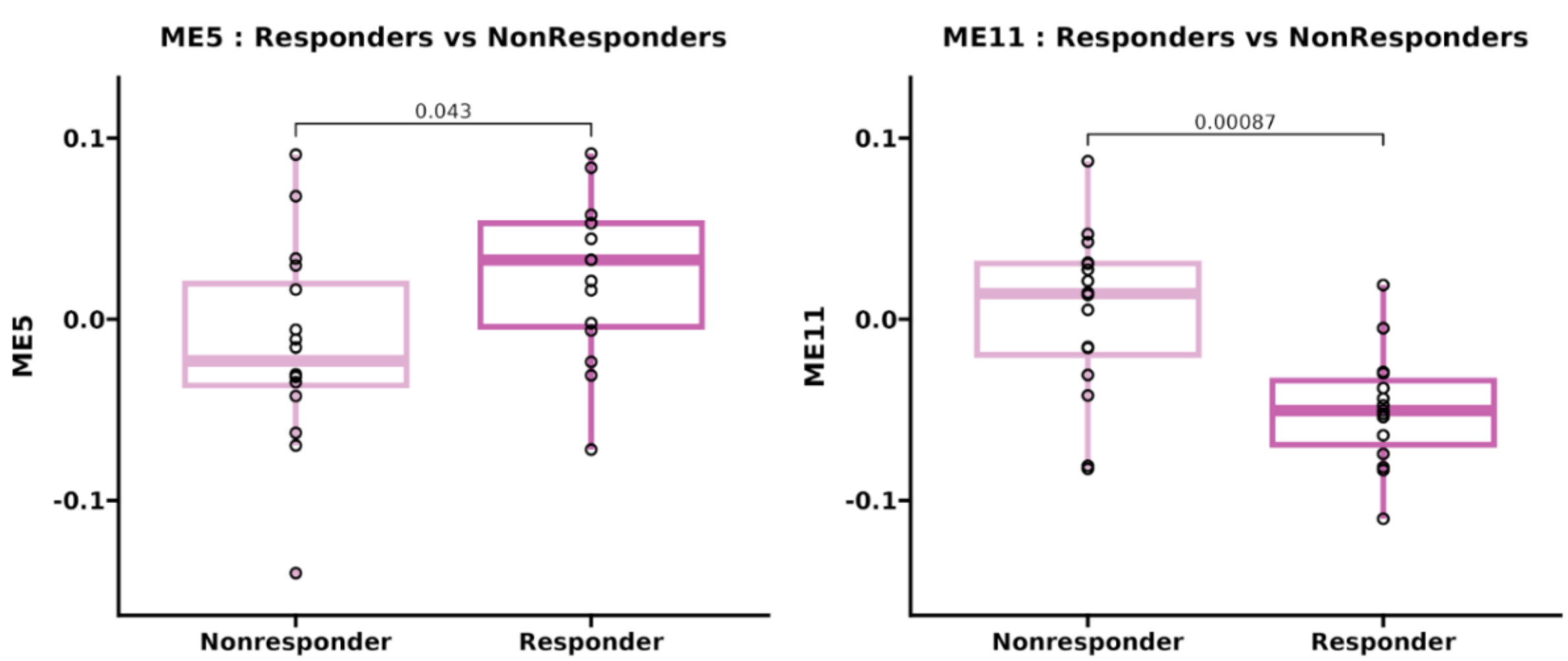

Supplemental Figure 15. Boxplots for significant baseline comparisons of responders to ECT.

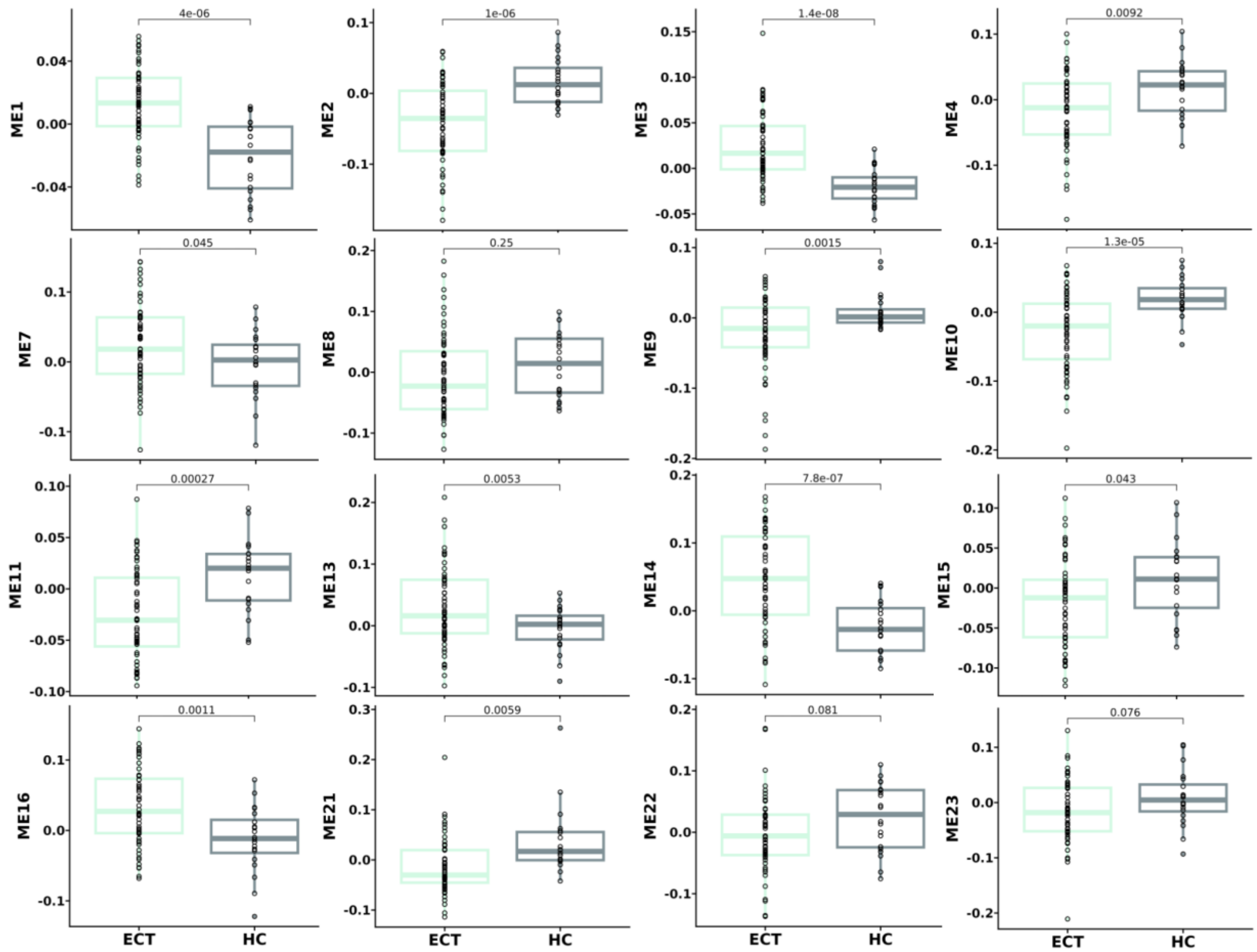

Supplemental Figure 16. Boxplots for significant eigengene differences between ECT patients and HC at baseline.
